# Molecular properties of a triazole–Ce(III) complex with antioxidant activity: structure, spectroscopy, and relationships with related derivatives. Influence of the ligands in the complex

**DOI:** 10.3389/fchem.2024.1450106

**Published:** 2024-11-06

**Authors:** M. Alcolea Palafox, Nataliya P. Belskaya, Lozan Todorov, Nadya Hristova-Avakumova, Irena P. Kostova

**Affiliations:** ^1^ Departamento de Química Física, Facultad de Ciencias Químicas, Universidad Complutense, Madrid, Spain; ^2^ Department of Technology for Organic Synthesis, Ural Federal University, Yekaterinburg, Russia; ^3^ Department of Chemistry, Faculty of Pharmacy, Medical University – Sofia, Sofia, Bulgaria; ^4^ Department of Medical Physics and Biophysics, Faculty of Medicine, Medical University of Sofia, Sofia, Bulgaria

**Keywords:** cerium(III) complex, 1,2,3-triazoles, antioxidant activity, structural relationships, infrared, Raman

## Abstract

A novel Ce(III) complex with the triazole ligand 2b, which presents four H-bonded sites with amino acids of the MMP-2 receptor, was synthesized. The experimental IR and Raman spectra of this Ce(III) complex were well-interpreted based on their comparison to the theoretical scaled spectra using the scaling equations determined by two procedures and four density functional theory (DFT) levels. Therefore, the structure predicted for the synthesized Ce(III) complex was clearly characterized and confirmed. The potential antioxidant action of this complex was compared with the analogous La(III) complex, and it was found that the coordination of ligand 2b with Ce(III) improves the ligand’s ability to participate in single-electron transfer (SET), as observed in the ABTS^·+^ assay, and this complex seems to scavenge the stable radical much more actively compared to its La(III) counterpart. Additionally, interactions with potassium superoxide and sodium hypochlorite indicate a high pro-oxidant behavior of the complex. The effects of different ligands on the geometric parameters, atomic charges, and molecular properties of the Ce(III) complex were analyzed at four DFT levels, and several relationships were clearly established. These relationships can facilitate the selection of new ligands with improved properties in the design of novel lanthanide–triazole carboxylate complexes with promising biological activity. The ligand size increase in the complexes facilitates the electronic transfer of negative charge, and the low HOMO (highest occupied molecular orbital)–LUMO (lowest unoccupied molecular orbital) energy gap indicates a large reactivity and low energy for their excitation.

## 1 Introduction

Coordination compounds with rare earth elements are being extensively developed for medical use (Goswami and Kostova, 2022; Lewandowski et al., 2005), especially as promising candidates for anticancer therapeutics (Fricker, 2006; Musib et al., 2023; Zhang et al., 2023). Lanthanide (Ln) complexes with a number of drugs (hymecromone, umbellipherone, mendiaxon, warfarin, coumachlor, and niffcoumar) have demonstrated cytotoxicity against the HL-60 myeloid cell line in preclinical studies (Chundawat et al., 2021). The lanthanide–texaphyrin complexes have advanced into clinical trials (Evens, 2004; Mody et al., 2001).

Special interest appears in the cerium complexes, which have been reported to have important pharmaceutical properties. For example, the cerium–humic acid complex has bacteriostatic potency, inhibiting the growth of several dangerous bacterial strains (Zhang et al., 2000), cerium–curcumin and cerium–quercetin complexes exhibit toxic effects against both breast and melanoma cancer cells used in photodynamic therapy (PDT) (Hosseinzadeh et al., 2021), cerium–ofloxacin and 2,2′-bipyridine complexes show antimicrobial and anticancer activities against breast and colon cell lines (Abd El-Hamid et al., 2019), and azamacrocyclic–cerium complexes promote the hydrolysis of the phosphodiester bond of supercoiled DNA (Feng et al., 2013). In addition, series of cerium(III) bipyridyl, phenanthroline, and related complexes have been reported with *in vitro* activity against cancer cell lines (Fricker, 2006). Despite the important properties of cerium complexes, they have received far less attention than lanthanum complexes, which is one of the reasons for their investigations in the present study.

The coordination chemistry of the lanthanoid metals is of interest in a variety of fields, such as molecular magnetic materials, catalysts, luminescent thermometers, or MRI contrast agents in bioimaging, nonlinear optics, and up-conversion materials (Bünzli, 2015; Li and Yan, 2020; Omodara et al., 2019; Zhang et al., 2021; Zhao et al., 2021), and the selection of a specific ligand for this coordination is an important and difficult task.

Ln(III) ions tend to form stable chemical bonds with organic compounds containing oxygen, nitrogen, or sulfur atoms (O, N, and S). Among the *N*-donor ligands, preferred heterocycles for Ln complexation are pyridine, pyrazole, tetrazole, and oxazole. There are well-known Ln(III) complexes with terpyridine, coumarin, acridine, porphyrin, quercetin, 1,10-phenanthroline, and benzotiazole (Fricker, 2006). Neutral *N*-1-substituted 1,2,3- triazoles have not been applied extensively as coordination units for Ln-complex formation because their binding affinities are not strong enough to overcome affinities with anions such as NO_3_^−^, F^−^, Cl^−^, and Br^−^ (Juwarker et al., 2009) or the hydration enthalpy of Ln(III) ions (Guo et al., 2023). 2-Substituted-1,2,3-triazoles have a more symmetrical electronic distribution that could not stabilize such constructions. However, the triazole core is quite attractive as it is biocompatible and tends to endow molecules with a broad profile of biological activities and diverse pharmacophore properties (Agalave et al., 2011; Bonandi et al., 2017; Bozorov et al., 2019; Giroud et al., 2018; Keri et al., 2015; Li et al., 2018; Zhang et al., 2009).

Triazoles are of particular interest to modern medicine for several reasons. In addition to having significant dipole moments, they are characterized by high stability in terms of redox reactions and hydrolysis. Nitrogen atoms in the structure can also participate in hydrogen bond formation, thus enhancing solubility (Dheer et al., 2017). The facility of the triazole ring to form these H-bonds is considered the main reason for its ability to enhance binding to target molecules (Raic-Malic and Mescic, 2015; Kumar et al., 2013; Saini and Dwivedi, 2013). Moreover, in a variety of studies (Slavova et al., 2020), triazoles have been utilized as linkers. Particularly, the triazole ring is not just a passive linker, and it contributes to biological activity by binding to target molecules via H-bonding and dipole interactions (Kolb and Sharpless, 2003). When combined with another compound, it can produce new biological effects, increasing the activity of molecules (Slavova et al., 2020). In terms of mechanisms of action, triazole-containing molecules can act as plant gibberellin inhibitors, carbonic anhydrase inhibitors, competitive inhibitors of *para-*aminobenzoic acid, cellular microtubule stabilizers, nucleoside analogs, etc.

Since there are many examples that have demonstrated the synergetic biological effect due to coordination with transition metals or lanthanides (Slavova et al., 2020; Gil-Moles and Concepción Gimeno, 2024; Podolski-Renić et al., 2024; Adhikari et al., 2024; Alcolea Palafox et al., 2023a), the 1,2,3-isomer has been selected because it has been shown to have the best properties (Agalave et al., 2011; Bonandi et al., 2017; Bozorov et al., 2019; Giroud et al., 2018; Slavova et al., 2020; Hrimla et al., 2021) as a ligand and is suitable to coordinate with the Ce(III) ion. In order to overcome the possible problem of instability of the desired complex, we employed a well-known approach based on attaching the side substituents and functionalities to heterocycles with the additional nucleophilic centers (Zafar et al., 2023; Hussain Sumrra et al., 2020).

**FIGURE 1:**
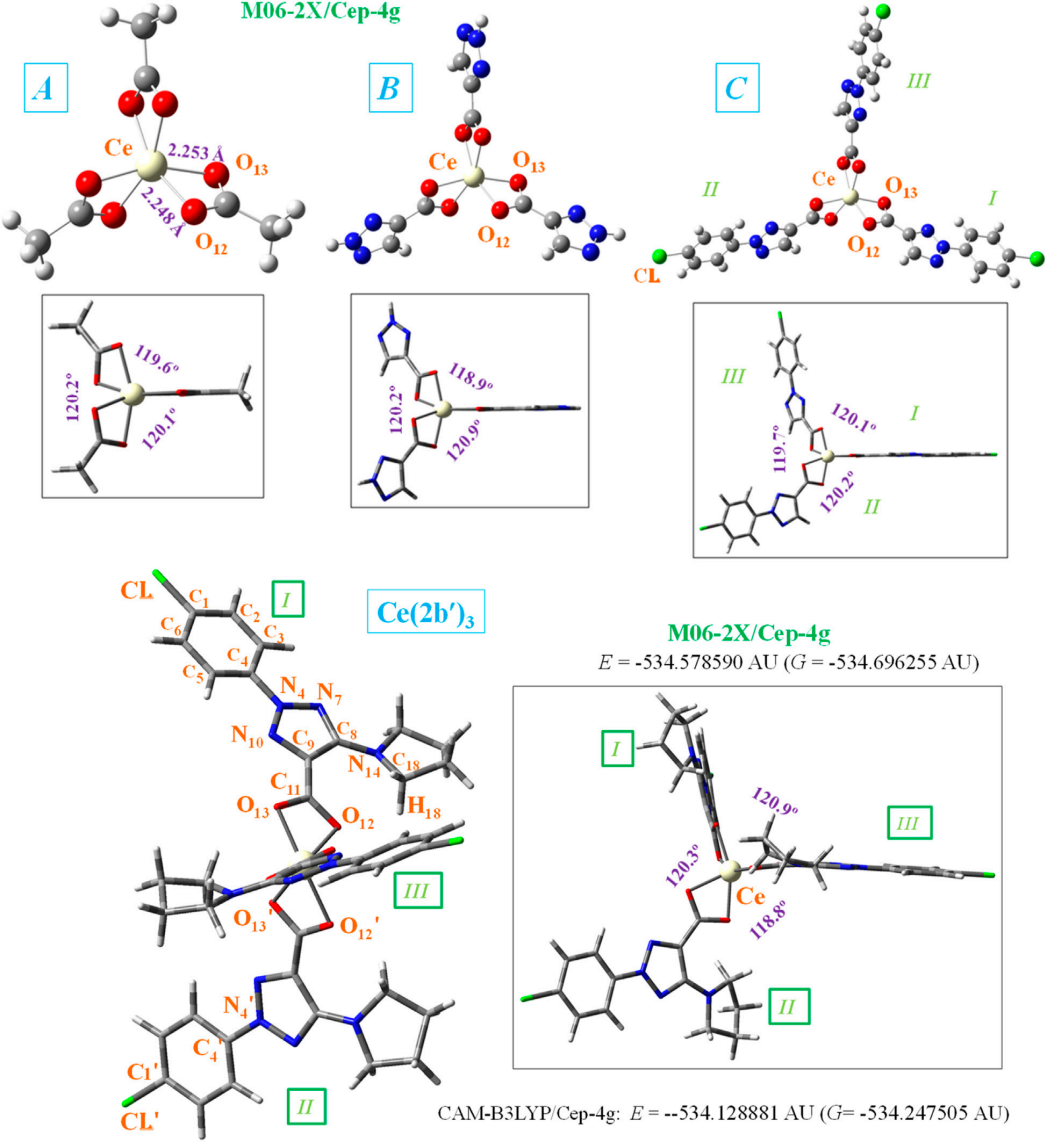
Front and lateral views of the optimized structure of four Ce(III) complexes at the CAM-B3LYP/Cep-4g and M06-2X/Cep-4g levels with the following ligands: acetate (A-complex), 1,2,3-triazole-4-carboxylate (B-complex), 2-(4-chlorophenyl)-2*H*-1,2,3-triazole-4-carboxylate (C-complex), and 2-(4-chlorophenyl)-5-(pyrrolidin-1-yl)-2*H*-1,2,3-triazole-4-carboxylate (2b′ ligand) (Ce (2b′)_3_ complex). The labeling of the most characteristic atoms, the total energy of the system (*E*) including zero-point correction, and the Gibbs free energy (*G*), as well as several bond length values, are also included. 1 AU = 2625.5 kJ/mol.

To facilitate the coordination of the triazole ring with the Ce(III) ion, this was carried out through the carboxylate group at C4 (C9 in Figure 1) of the triazole ring. In addition, aryl and pyrrolidine groups were bonded to the triazole ring to provide liposolubility to facilitate cell penetration. A chlorine atom was also attached to the aryl group, constituting the 2-(4-chlorophenyl)-5-(pyrrolidin-1-yl)-2*H*-1,2,3-triazole-4-carboxylate ligand. This triazole ligand has been labeled as 2b (sodium salt) and 2b′ (its anionic form) (Alcolea Palafox et al., 2023b), and such notation will be used here. The ligand 2b has been previously studied from the structural, spectroscopic, and molecular docking points of view (Alcolea Palafox et al., 2023b; Safronov et al., 2023; Palafox et al., 2023). The cerium cation is now coordinated with this ligand via the carboxylate groups, linked in a three-dimensional coordination complex, as shown in Figure 1. This arrangement is similar to that corresponding to the La(III) complex first obtained by us (Alcolea Palafox et al., 2023a), as well as in complexes of other carboxylic acid derivatives (Zhao et al., 2021). The *in vitro* behavior of this new complex toward a wide range of RS-generating model systems revealed potential therapeutic applications as hydroxyl scavengers and potential pro-oxidants (Alcolea Palafox et al., 2023a; Alcolea Palafox et al., 2023b).

Because the most accurate of the quantum chemical methods are still too expensive to apply in routine research, density functional theory (DFT) has emerged during the past decades as a powerful methodology for the simulation of chemical systems. DFT methods are less computationally demanding than other theoretical methods with a similar accuracy, or even better in some cases, such as the theoretical prediction of vibrational spectra (Palafox, 2018). They are being able to include electron correlation in the calculations at a fraction of time of post-Hartree–Fock methodologies. Therefore, these DFT methods have a widespread application and are becoming increasingly popular.

Among the multiple applications of DFT methods, one based on the molecular docking approach has been used against SARS-CoV-2 (Subbiah et al., 2022) and promising drug targets for cancer (Noreen et al., 2023; Sert et al., 2020). However, our interest in the present manuscript is related to the interpretation of the spectra, which have been developed specifically over the last decade. Although they have been used for the assignment of the UV/vis (Sert et al., 2020; Hassan et al., 2023; Barim and Akman, 2022) and ^1^H and ^13^C NMR spectra (Sert et al., 2020; Rajaraman et al., 2022), in the present work, we focus our attention only on the IR and Raman vibrational spectra (Dodoo et al., 2023; Evangalin et al., 2018; Singh et al., 2023). Vibrational spectroscopy is one of the most powerful techniques for the characterization of medium-sized molecules. An accurate interpretation of vibrational experimental spectra using DFT methods is essential in many fields of chemistry and has become an important part of spectrochemical and quantum chemical investigations (Palafox, 2018; Alcolea Palafox, 2017).

However, the proper assignment of spectra is often not straightforward, and a large overestimation of the calculated vibrational wavenumbers is expected. This overestimation (which may be due to many different factors that are usually not even considered in the theory, such as anharmonicity, errors in the computed geometry, Fermi resonance, and solvent effects) can be significantly reduced with the use of transferable empirical parameters for the calculated wavenumbers. The use of suitable DFT quantum-chemical methods and scaling procedures remarkably reduces the risk in the assignment and can accurately determine the contribution of different modes in an observed band. Now, these procedures appear to be extensively used in the journals of vibrational spectroscopy, and we applied them in this study.

Now, the two main aims of the current investigation are as follows: 1) the first aim was to synthesize and characterize a newly synthesized cerium(III) complex of the 2-(4-chlorophenyl)-5-(pyrrolidin-1-yl)-2*H*-1,2,3-triazole-4- carboxylate (2b′) ligand and study its potential antioxidant activity. This ligand appears to be of great interest as it presents H-bonded sites with the matrix metalloproteinase-2 (MMP-2) receptor (Palafox et al., 2023), which is one of the main models for angiogenesis and tumor development (Jezierska and Motyl, 2009). The product develops as an amorphous precipitate and does not provide crystals suitable for X-ray molecular structure determination. Since the crystal structure data on this newly synthesized Ce (2b′)_3_ coordination complex are not available, in such cases, theoretical calculations are very informative and useful. Moreover, theoretical approaches for the determination of geometrical parameters, vibrational frequencies with accurate scaling procedures, hydrogen bond strengths, model metal(III)–ligand bonding, and conformation of the molecular structure proposed at a high level of theory are very helpful for extracting reliable structural information. Therefore, these theoretical approaches were carried out in this study, and they helped predict correctly the metal coordination polyhedron of the studied complex.

The potential antioxidant activity of the Ce(III) complex was assessed utilizing multiple reactive species (RS)-generating model systems. Therefore, the impact of the Ce(III) complex on the physiologically important hydroxyl radicals (two different experiments), superoxide radical ions, and hypochlorite ions was investigated. Potential mechanisms of action—single-electron transfer (SET) and hydrogen atom transfer (HAT)— will be elucidated with the aid of the 2,2′-azino-bis(3-ethylbenzothiazoline-6-sulfonic) free radical (ABTS^·+^) and 2,2-diphenyl-1-picrylhydrazyl radical (DPPH^·^) assays. The complex’s behavior in these model systems at the highest concentration tested was compared to a well-established positive control: 6-hydroxy-2,5,7,8-tetramethylchroman-2-carboxylic acid (Trolox) (Goswami and Kostova, 2022). 2) Another aim was to reveal the impact of structural fragments on the features of the geometry and electronic structure of the triazole complex. For this purpose, three simpler complexes, namely, A- (acetate), B- (1,2,3-triazole-4-carboxylate), and C- (2-(4-chlorophenyl)-2*H*-1,2,3-triazole-4-carboxylate), were visualized and used as models for quantum mechanical chemical calculations (Figure 1). Therefore, new relationships with different ligands were established to find better properties, which will facilitate the selection of new ligands for further design of new complexes with improved properties.

## 2 Materials and methods

### 2.1 Materials

All compounds used were of pro analysis grade from Sigma-Aldrich. Ethanol (95%) and bi-distilled water were utilized for the preparation of the solution. Trichloroacetic acid (TCA), thiobarbituric acid (TBA), and K–Na-phosphate buffered saline (PBS) solutions were required for the deoxyribose degradation assay, according to that reported in Alcolea Palafox et al. (2023a). 3-(4,5-Dimethylthiazol-2-yl)-2,5-diphenyltetrazolium bromide (MTT) was required, as well as other reagents for the Fenton reaction MTT assay. Participation in hydrogen atom transfer (HAT) reactions was elucidated following the protocols (Kedare and Singh, 2011; Molyneux, 2004). The participation of the Ce(III) complex in single-electron transfer (SET) reactions was determined according to that reported in Erel (2004a) and Erel (2004b). Luminol-dependent chemiluminescence (LDCL) was utilized to assess scavenging of (O_2_^⋅-^) radicals.

### 2.2 Experiment

The Ce(III) complex was synthesized from Merck’s Ce(NO_3_)_3_·6H_2_O salt (99%) and the 2b ligand (Safronov et al., 2023). The procedure is similar to that followed in the synthesis of the La (2b′)_3_ complex (Alcolea Palafox et al., 2023a). The chemical composition of the newly obtained Ce(III) complex was characterized via elemental analysis. The binding mode in the Ce(III) complex was confirmed via vibrational spectroscopy. The synthesized Ce (2b′)_3_ complex was investigated as KBr pellet at room temperature for the IR spectrum, which was plotted in the 3,900–400 cm^–1^ region utilizing a Bruker IFS25 FTIR spectrophotometer. The Raman spectrum was registered in the 4,000–0-cm^−1^ range using a Horiba Jobin Yvon’s spectrometer.

UV-vis spectra were recorded using a Shimadzu UV-1601 spectrophotometer. For LDCL measurements, an LKB 1251 luminometer (Bio-Orbit, Turku, Finland) was set at 37°C and connected to an IT-type computer via a serial interface. Three samples have been tested in each concentration. Averages and standard deviations were calculated. Relative changes within the limits of experimental errors were not discussed.

### 2.3 Methods

Stock solutions of the investigated ligand 2b (1*·*10^−3^ M) and its complex Ce (2b′)_3_ (1*·*10^−4^ M) were prepared using bi-distilled water. In order to test the molarities of 1*·*10^−6^ M or lower, these solutions were further diluted as necessary. A 1*·*10^−3^ M stock solution of Trolox in PBS was prepared. The activity of the cerium complex was compared to that of Trolox at the highest concentration tested (3*·*10^−5^ M).

For each tested molarity, three replicates were run, each representing an individual datapoint. Average values and standard deviations were calculated. The impact of the tested compounds on the RS-generating model systems was statistically verified via one-way ANOVA, followed by the Bonferroni post-test. Differences with *p* < 0.05 were considered statistically significant. Relative changes within the limits of the experimental error were not discussed.

#### 2.3.1 Deoxyribose degradation assay

Hydroxyl radicals (OH^·^) were generated via UV-induced water radiolysis (Burns and Sims, 1981) through a modified protocol (Halliwell et al., 1987). The TBA assay was applied to assess the degradation of 2-deoxyribose (2-DR) to malondialdehyde (MDA). The 2-DR degradation degree is calculated as radical-scavenging activity (RSA) by Equation 1:

$$\text{RSA},\%=\frac{A_{\text{control}}-\left(A_{\text{sample}}-A_{\text{blank}}\right)}{A_{\text{control}}}\cdot 100,$$ (1)

where “control” sample excludes the Ce(III) complex. “Sample” is composed of 2-DR (0.5 mL) + Ce(III) complex solution + PBS (up to 5 mL). This 5-mL sample was irradiated using UV, and TBA and TCA were added following the procedure described in Alcolea Palafox et al. (2023a).

The *in vitro* scavenging activity of the sodium salt of the ligand (2b′) and its La(III) complex in the presence of several free radical-generating model systems was discussed in Alcolea Palafox et al. (2023a). Herein, the behavior of 2b and its Ce(III) complex on RS, generated by six model systems, is revealed and compared to that of the respective La(III) complex.

#### 2.3.2 ABTS, DPPH, and Fenton reaction MTT assays

Three types of samples, namely, “blank,” “control,” and “sample,” were tested using the kinetic function of the apparatus with the lag time of 10 s and measuring time of 600 s. In each sample composition, 1.0 mL is the total volume in ABTS, whereas it is 2.0 mL in DPPH and MTT assays. Results are presented as RSA, which is similar to the 2-deoxyribose degradation assay, as shown in Table 1.

**TABLE 1:** Fenton reaction with ABTS, DPPH, and MTT assay sample compositions. Values are in mL.

Assay	Reagent	Blank	Control	Sample
ABTS	Tested compound	0.1	-	0.1
	R1	0.86	0.86	0.86
	R2	-	0.04	0.04
	Bi-distilled water	0.04	0.1	-
DPPH	Tested compound	0.2	-	0.2
	DPPH	-	1.8	1.8
	Ethanol	1.8	-	-
	Bi-distilled water	-	0.2	-
MTT	Tested compound	0.2	-	0.2
	MTT	0.2	0.2	0.2
	Fe^2+^/H_2_O_2_/Na_2_-EDTA	-	0.1	0.1
	Ascorbic acid	-	0.1	0.1
	Bi-distilled water	up to 2.0	to 2.0	tо 2.0

ABTS: Prior to experimentation, two reagents (R1 and R2) are prepared (Erel, 2004a), where R1 is Na-acetate buffer with pH = 5.8, while R2 is composed of ABTS dissolved in Na-acetate buffer with pH = 3.8, along with the addition of H_2_O_2_ to form ABTS^·+^ radical. Absorbance was determined at 660 nm.

DPPH: It was performed according to literature data (Kedare and Singh, 2011; Molyneux, 2004; Chrzczanowicz et al., 2008), and where DPPH^·^ absorbance changes were measured at λ = 517 nm.

MTT: The reduction in MTT to formazan by Fenton-generated OH^·^ was investigated. Ascorbic acid increases the formation of OH^·^. Formazan production causes an increase in the absorbance at λ = 578 nm.

#### 2.3.3 LDCL in the presence of KO_2_ or NaClO

*With KO*_*2*_, the “control” composition (1.0 mL) contains luminol (0.05 mL) + KO_2_ (0.05 mL) + PBS. The “sample” composition (1.0 mL) contains the Ce(III) complex + luminol (0.05 mL) + KO_2_ (0.05 mL) + PBS. The kinetic mode of the apparatus was utilized. Measurements are taken with 2-s delay from the total of 10 s. Data are presented as the CL-SI index by Equation 2:

$$CL-SI,\%=\frac{I_{sample}}{I_{control}}\cdot 100.$$ (2)

In the absence of KO_2_, the background signal has been withdrawn from all data recorded.

*With NaClO*, the “control” composition (1.0 mL) contains NaClO (0.15 mL) + luminol (0.05 mL) + PBS. The “sample” composition (1.0 mL) contains Ce(III) complex + luminol (0.05 mL) + NaClO (0.15 mL) + PBS. The background signal is recorded in the absence of hypochlorite and is withdrawn from all data recorded. Data are also shown as the CL-SI index.

### 2.4 Computational details

Four theoretical levels were used for the optimization of all Ce(III) complexes. Density functional theory (DFT) methods (Seminario and Politzer, 1995) were chosen for this purpose since they have provided accurate vibrational wavenumber values in biomolecules that are in good agreement with the experimental values and in better agreement compared to those determined by the MP2 method (Palafox, 2018). Among these DFT methods, the Minnesota M06-2X functional was the most preferred because it is one of the best meta-generalized gradient functionals to analyze dispersion bound in large systems (Riley and Hobza, 2011; Riley et al., 2010), especially in biomolecules with non-covalent weak interactions, like those included in the present work. Moreover, this method has also shown a large applicability in chemistry (Zhao and Truhlar, 2011).

The B3LYP functional has been chosen second because it has yielded excellent results in the computation of the IR and Raman wavenumbers of biomolecules and is better than other DFT methods (Palafox, 2018; Alcolea Palafox, 2000). In particular, with the Ce(III) ion, it performs slightly better than other hybrid functionals (Kullgren et al., 2010). However, the B3LYP functional alone is not appropriate for reproducing systems with non-covalent weak interactions, like those observed with our complexes; therefore, the spectra obtained differ from the experimental spectra. For this reason, the D3-B3LYP and the CAM-B3LYP methods (Yanai et al., 2004), which combine the hybrid qualities of B3LYP and the long-range correction (Tawada et al., 2004), were also used to improve the calculated structural parameters. The Cep-4g basis set is the only set that appears available in the Gaussian-16 program package for cerium (Frisch et al., 2016). Although it is a very small basis set, the vibration spectra obtained with this basis set can be well correlated with the experimental spectra, and therefore, a good characterization of the synthesized Ce (2b′)_3_ complex was carried out. Even with the smaller Ce(III) complexes labeled as A, B, and C as shown in Figure 1, the three DFT methods required a charge +1 on the whole system. With the neutral charge 0 on the system, the DFT methods used in the Gaussian-16 program package does not run, indicating an inconsistency in the ground state calculations (multiplicity = 1). The authors consider this unusual since the three positive charges on the Ce ion are compensated by three 2b′ anions. Notably, this could be due to the default of the Cep-4g basis for the Ce ion. Despite the fact that the effect on the organic ligands is very slight and their calculated spectra are in accordance with the experimental spectra, various Ce(III) and Ce(IV) complexes with organic molecules have been reported (Chundawat et al., 2021; Evens, 2004; Das et al., 2014; Levin et al., 2016). The UNIX version of the Gaussian-16 program with standard parameters was operated in the Brigit super-computer of the Complutense University of Madrid.

All optimized complexes showed positive wavenumbers, indicating a minimum in the potential energy surface. For this task, harmonic wavenumber computations were performed at the same level of the corresponding optimization process.

#### 2.4.1 Scaling the wavenumbers

The calculated wavenumbers by the theoretical DFT methods appear overestimated due to different reasons (Alcolea Palafox, 2019). To correct this overestimation, different scaling procedures have been reported for each specific method and basis set (Alcolea Palafox, 2000; Alcolea Palafox, 2019), obtaining a noticeable improvement in the wavenumbers. To get an accurate assignment of the experimental bands, the scaling is the normal procedure followed by the authors. Among the different procedures available, the linear scaling equation (LSE) (Equations 3–6) and polynomic scaling equation (PSE) (Equations 7–10) procedures appear as the most appropriate, and therefore, they were used in the present study. The specific equations utilized here for the four levels of computation are as follows:

$$\text{LSE procedure}:\nu^{\text{scal}}=195.7+0.8706\;\nu^{\text{cal}}\text{ at }B3\text{LYP}/\text{Cep}‐4g\text{ level},$$ (3)

$$\nu^{\text{scal}}=194.0+0.8714\;\nu^{\text{cal}}\text{ at }D3‐B3\text{LYP}/\text{Cep}‐4g\text{ level},$$ (4)

$$\nu^{\text{scal}}=184.0+0.8646\;\nu^{\text{cal}}\text{ at CAM}‐B3\text{LYP}/\text{Cep}‐4g\text{ level},$$ (5)

$$\nu^{\text{scal}}=185.0+0.8613\;\nu^{\text{cal}}\text{ at }M06‐2X/\text{Cep}‐4g\text{ level},$$ (6)

$$\text{PSE procedure}:\nu^{\text{scal}}=90.6+1.0305\;\nu^{\text{cal}}\;‐\;0.0000403\cdot \;{\left(\nu^{\text{cal}}\;\right)}^{2}\text{ at }B3\text{LYP}/\text{Cep}‐4g\text{ level},$$ (7)

$$\nu^{\text{scal}}=92.7+1.0253\;\nu^{\text{cal}}\;‐\;0.0000388\cdot \;{\left(\nu^{\text{cal}}\;\right)}^{2}\text{ at }D3‐B3\text{LYP}/\text{Cep}‐4g\text{ level},$$ (8)

$$\nu^{\text{scal}}=78.7+1.0216\;\nu^{\text{cal}}\;‐\;0.0000390\cdot \;{\left(\nu^{\text{cal}}\;\right)}^{2}\text{ at CAM}‐B3\text{LYP}/\text{Cep}‐4g\text{ level},$$ (9)

$$\nu^{\text{scal}}=78.0+1,0204\;\nu^{\text{cal}}\;‐\;0.0000394\cdot \;{\left(\nu^{\text{cal}}\;\right)}^{2}\text{ at }M06‐2X/\text{ Cep}‐4g\text{ level}.$$ (10)

## 3 Results and discussion

**SCHEME 1:**
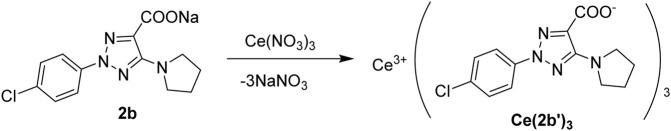
Synthesis of the Ce(III) complex.

The *N*-2-aryl-triazole ligand 2b was prepared according to the procedures reported in the literature (Alcolea Palafox et al., 2023a; Safronov et al., 2023; Palafox et al., 2023). The complex Ce (2b′)_3_ was synthesized by the interaction of triazole sodium salt 2b with Ce(NO_3_)_3_·6H_2_O at a molar ratio of 3:1 in the water solution (Scheme 1) with a yield of 85% and characterized via elemental analysis and vibrational spectroscopy.

The elemental analysis of the Ce(III) complex of 2-(4-chlorophenyl)-5-(pyrrolidin-1-yl)-2*H*-1,2,3-triazole-4-carboxylic acid: (% calculated/found): Ce (2b′)_3_^·^ 2H_2_O: C: 44.61/45.02; H: 4.00/3.78; N: 16.01/15.68; and H_2_O: 3.43/3.98; Ce: 13.35/12.95, where 2b′ = C_13_H_12_N_4_O_2_Cl^−^.

The binding mode of the ligand to Ce(III) ions was elucidated by recording the IR and Raman spectra of the complex, as compared with those of the free ligand and the theoretical predictions. The vibrational fundamentals from the IR and Raman spectra were analyzed by comparing these modes with literature sources in combination with the results derived from DFT calculations (i.e., harmonic vibrational wavenumbers and their Raman scattering activities) for the ligand and Ce(III) complex.

### 3.1 Molecular structure of the cerium complex

The cerium(III) ion, similar to other lanthanide ions, appears to coordinate well with oxygen atoms rather than nitrogen atoms (Peica et al., 2006). This could be due to the large flexibility of the carboxylic oxygens, which facilitates bonding with Ce^3+^. Therefore, the starting geometry to be optimized was that with the Ce(III) ion coordinated through the COO^−^ group with three ligands. For simplicity, as shown at the bottom of Figure 1, the total energy (*E*), which includes the ZPE (zero-point vibrational energy) correction, and the Gibbs energy (*G*) value were only shown for the Ce (2b′)_3_ complex and with the CAM-B3LYP and M06-2X methods. Because the optimized structure by the B3LYP and D3-B3LYP methods appears noticeably distorted, they are included in Supplementary Figures S1, S2. This distortion is due to the lack of long-range interactions of B3LYP for stabilizing the complex to a symmetric arrangement.

In the complex formation with the cerium ion, the CO bonds of the ligands are lengthened, as compared to the free form or in a dimer form (Alcolea Palafox et al., 2023b), which causes a slight shortening of the C_9_–C_11_ bond length. This feature in the triazole ring leads to a decrease in the N_7_ = C_8_ and C_9_ = N_10_ double-bond character (an increase in their bond lengths) and shortening of the neighbor N_4_–N_10_ and C_8_–N_14_ bonds. It is noted that the lengthening of these bond lengths leads to more rotated triazole substituents, and thus, they can interact more easily with other ligands, especially through the pyrrolidine ring. Therefore, with cerium binding, the triazole ring bonds and angles are slightly modified, which consequently results in the modification of their molecular properties.

Different ligands have little impact on the almost symmetric arrangement with the Ce(III) ion, as shown in Figure 1. Therefore, all methods and complexes place these ligands at angles (C_11_···Ce···C′_11_ and C′_11_···Ce···C″_11_) very close to 120.0° and with very little rotation, with C_9_–C_11_···C′_11_–C′_9_ and C′_9_–C′_11_···C″_11_–C″_9_ torsional angles close to 0°, with the exception of the calculated values in the Ce (2b′)_3_ complex by the M06-2X method, −18.0° and 16.7°, respectively. As expected, the main differences appear in the coordination distances between the carboxylate oxygens and Ce(III) ion, which significantly affects the neighboring O-C_11_ (slightly, for example, 1.434, 1.434, 1.436, and 1.437 Å) and (more prominently: 1.657, 1.587, 1.583, and 1.576 Å) C_11_–C_9_ bond lengths. These differences are presented in Table 2, which includes several selected optimized geometrical parameters in one of the ligands (labeled as *I*) at three DFT levels. The notation used for labeling the atoms is from that reported in the 2b′ ligand (Safronov et al., 2023). Large differences also appear among the three DFT levels used, with the values by CAM-B3LYP closer to M06-2X than to B3LYP.

**TABLE 2:** Several selected optimized geometrical parameters calculated with different DFT methods and the Cep-4g basis set in the Ce(III) complexes shown in Figure 1. Bond lengths (r) are in Å, and bond angles and dihedral angles (∠) are in degrees. The data were from ligand *I*.

	A complex			B complex			C complex			Ce(2b′)_3_			
Parameter	B3LYP	CAM-B3LYP	M06-2X	B3LYP	CAM-B3LYP	M06-2X	B3LYP	CAM-B3LYP	M06-2X	B3LYP	D3-B3LYP	CAM-B3LYP	M06-2X
r (C_9_–C_11_)	1.657	1.648	1.638	1.587	1.581	1.575	1.583	1.577	1.571	1.576	1.570	1.563	1.556
r (C=O_12_)	1.434	1.423	1.413	1.434	1.422	1.410	1.436	1.423	1.413	1.437	1.437	1.425	1.415
r (C=O_13_)	1.434	1.423	1.412	1.435	1.423	1.414	1.436	1.423	1.413	1.442	1.441	1.433	1.425
r (Ce–O_12_)	2.287	2.256	2.248	2.290	2.260	2.261	2.288	2.257	2.253	2.304	2.292	2.264	2.260
r (Ce–O_13_)	2.286	2.255	2.253	2.282	2.251	2.240	2.281	2.251	2.245	2.275	2.272	2.231	2.224
∠(C_9_–C_11_ = O_12_)	123.7	123.9	123.4	122.2	122.4	122.0	122.1	122.4	121.7	125.9	125.4	126.6	125.5
∠(O=C=O)	112.5	112.1	113.2	114.2	113.8	114.8	114.2	113.7	114.8	113.7	113.9	112.7	113.8
∠(C=O_12_–Ce)	92.3	92.3	91.9	90.9	91.0	90.3	90,9	91.1	90.5	90.9	90.8	91.1	90.4
∠(C=O_13_–Ce)	92.3	92.4	91.7	91.2	91.4	91.0	91.2	91.3	90.8	91.9	91.5	92.2	91.6
∠(O_12_–Ce–O′_13_)	153.9	155.3	100.5	154.3	155.5	100.5	153.8	155.3	101.7	152.4	153.3	154.8	99.7
∠(O_13_–Ce–O′_12_)	102.2	100.8	102.1	102.7	101.2	100.1	103.1	101.3	100.4	104.5	103.4	101.6	99.9
∠(C_11_–O_12_···O′_12_–C′_11_)	57.6	60.6	−98.1	56.9	60.5	−98.8	55.9	59.8	−99.1	56.4	59.6	61.7	−103.5
∠(C_9_–C_11_··· C′_11_–C′_9_)	−0.1	−0.2	0.5	0.2	0.0	1.8	0.0	0.0	2.7	−0.2	−1.1	0.0	−18.0
∠(C′_9_–C′_11_··· C″_11_–C″_9_)	0.1	0.3	−0.5	2.1	2.6	0.2	0.0	0.1	1.0	−0.1	−1.1	−0.1	16.7
∠(C_11_···Ce··· C′_11_)	120.1	120.2	119.6	120.2	120.2	118.9	119,9	120.1	120.1	120.0	119.9	119.8	120.3
∠(C′_11_···Ce··· C″_11_)	120.0	119.6	120.2	119.0	119.0	120.9	120.1	120.0	119.7	119.8	119.8	119.9	118.8

The optimized structure of the aryl ring in complexes C- and Ce (2b′)_3_ is full planar at all three DFT levels used. It is almost coplanar with the triazole ring, with the C_5_-C_4_-N_4_-N_10_ torsional angle of ca. −0.4° in the C complex and slightly larger due to the effect of the pyrrolidine ring in the Ce (2b′)_3_ complex, −4.7° by B3LYP and −2.3° by M06-2X. Similar values are also observed in the isolated ligands of C and Ce (2b′)_3_, which indicates a very small impact of the Ce(III) ion in this coplanarity.

The triazole ring is also full planar in B and C complexes at all three DFT levels and with torsional angle values lower than 0.5° in Ce (2b′)_3_. By contrast, the pyrrolidine substituent appears out-of-plane, as expected, and out-of-coplanarity with the triazole ring plane, C_9_-C_8_-N_14_-C_18_ = −20.2° by M06-2X and 16.6° by CAM-B3LYP. However, this value is noticeably lower than that calculated in the 2b ligand alone by the M06-2X method, −40.3°. This can be explained by the strong intramolecular H-bond O_12_···H_18_ between the carboxylate oxygen O_12_ and the pyrrolidine hydrogen H_18_, 1.711 Å vs. 2.605 Å in the Ce (2b′)_3_ complex. This H-bond forces the rotation of the pyrrolidine ring.

In the Ce (2b′)_3_ complex, the C_11_ carbon atom appears slightly rotated related to the triazole ring plane, with this rotation being slightly lesser with O_12_ than with O_13_. Therefore, the torsional angle C_8_-C_9_-C=O_12_ with O_12_ has a smaller value of −1.7° by M06-2X (4.8° by CAM-B3LYP) vs that with O_13_, N_10_-C_9_-C=O_13_ of −8.2° (8.9° by CAM-B3LYP). This difference is due to a weak intramolecular H-bond O_12_···H_18_, 2.605 Å by M06-2X (2.332 Å by CAM-B3LYP), but it has not been observed in complexes B and C which lack the pyrrolidine ring. Therefore, in complexes B and C, the carboxylate group appears coplanar to the triazole ring plane and with the same N_10_-C_9_-C=O_13_ and C_8_-C_9_-C=O_12_ torsional angle values of −0.2°. This intramolecular H-bond in the Ce (2b′)_3_ complex is also the main reason for the difference in Ce–O_12_ and Ce–O_13_ coordination distance values, especially in the C–O_12_ and C–O_13_ bond lengths. They are different in the Ce (2b′)_3_ complex but same in A, B, and C complexes.

Different ligands also have little influence on the calculated bond angles, such as C=O_12_–Ce (90.3° by M06-2X in the B complex and 90.4° in the Ce (2b′)_3_ complex), the C=O_13_–Ce angle (91.0° in the B complex and 91.6° in the Ce (2b′)_3_ complex), as well as the OCO, O_12_–Ce–O′_13_, and O_13_–Ce–O′_12_ angles, with differences lower than 2°, as shown in Table 2.

Significant differences are observed if we compare complexes Ce (2b′)_3_ and La (2b′)_3,_ published previously by Alcolea Palafox et al. (2023a). For example, the lengths of the C–O and C–C bonds surrounding the metal cation in the cerium complex are noticeably shorter (approximately 7%–13% by M06-2X), whereas the La^3+^–O bonds are noticeably shorter (approximately 10%–11%). These differences in the spatial structures of the complexes may affect their behavior in biological systems and the magnitude or selectivity of their biological effects.

### 3.2 APT atomic charges and relationships established

In the complexes under the theoretical study, the oxygen atoms have the highest negative charge, as expected, making them appear to be the most reactive. Due to their high reactivity, it is also expected that they would play a key role in the H-bonding of these complexes to amino acids of the cancer cell proteins. N_4_ and N_14_ nitrogen atoms also have a large negative charge, although lower than that of the oxygen atoms, and therefore, these atoms are also expected to participate in the biological activity of the synthesized complexes under the investigation. N_7_ nitrogen has a very small negative charge, and N_10_ has a small positive charge. Thus, these nitrogen atoms should be less active in potential interactions with biological targets.

C_8_ and C_11_ carbon atoms have a high positive charge due to the fact that they are bonded with highly negatively charged atoms. In the calculations performed, the Ce(III) ion appears positively charged around 3–4*e* depending on the DFT method used and complex studied, as shown in Table 3. It is smaller by B3LYP (between 2.3*e* and 3.7*e*) and larger by CAM-B3LYP and M06-2X methods (between 2.4*e* and 4.5*e*). CAM-B3LYP and M06-2X methods yield similar results. From A to Ce (2b′)_3_ complexes, the cerium charge is increased. This could be explained by a better charge distribution on the cerium ion in larger ligands than in smaller ligands, such as in A complex.

**TABLE 3:** Atomic polar tensor charges calculated with different DFT methods and the Cep-4g basis set in the Ce(III) complexes shown in Figure 1. The ligand APT values are only shown.

	A complex			B complex			C complex			Ce(2b′)_3_			
Atom	B3LYP	CAM-B3LYP	M06-2X	B3LYP	CAM-B3LYP	M06-2X	B3LYP	CAM-B3LYP	M06-2X	B3LYP	D3-B3LYP	CAM-B3LYP	M06-2X
**Ce**	2.366	2.814	2,752	2.982	3.464	3.436	3.657	4.147	4.117	2.844	3.056	4.457	4.494
N_4_	-	-	-	−0.596	−0.608	−0.625	−0.210	−0.440	−0.383	−0.058	−0.109	−0.466	−0.430
N_7_	-	-	-	−0.013	−0.018	−0.031	−0.111	−0.076	−0.105	−0.099	−0.109	−0.124	−0.133
C_8_	-	-	-	−0.071	−0.090	−0.079	0.021	−0.093	−0.072	0.444	0.453	0.596	0.610
C_9_	−0.450	−0.461	−0.512	−0.682	−0.664	−0.695	−1.065	−1.042	−1.100	−1.048	−1.096	−1.342	−1.392
N_10_	-	-	--	0.253	0.262	0.276	0.335	0.443	0.472	0.105	0.157	0.411	0.426
C_11_	0.830	0.831	0.810	1.459	1.446	1.441	1.954	1.871	1.900	1.458	1.591	2.071	2.134
O_12_	−0.733	−0.819	−0.794	−0.921	−0.995	−0.989	−1.205	−1.241	−1.260	−0.909	−0.974	−1.323	−1.363
O_13_	−0.747	−0.823	−0.797	−0.937	−1.011	−0.992	−1.167	−1.224	−1.201	−0.925	−1.001	−1.380	−1.413

**FIGURE 2:**
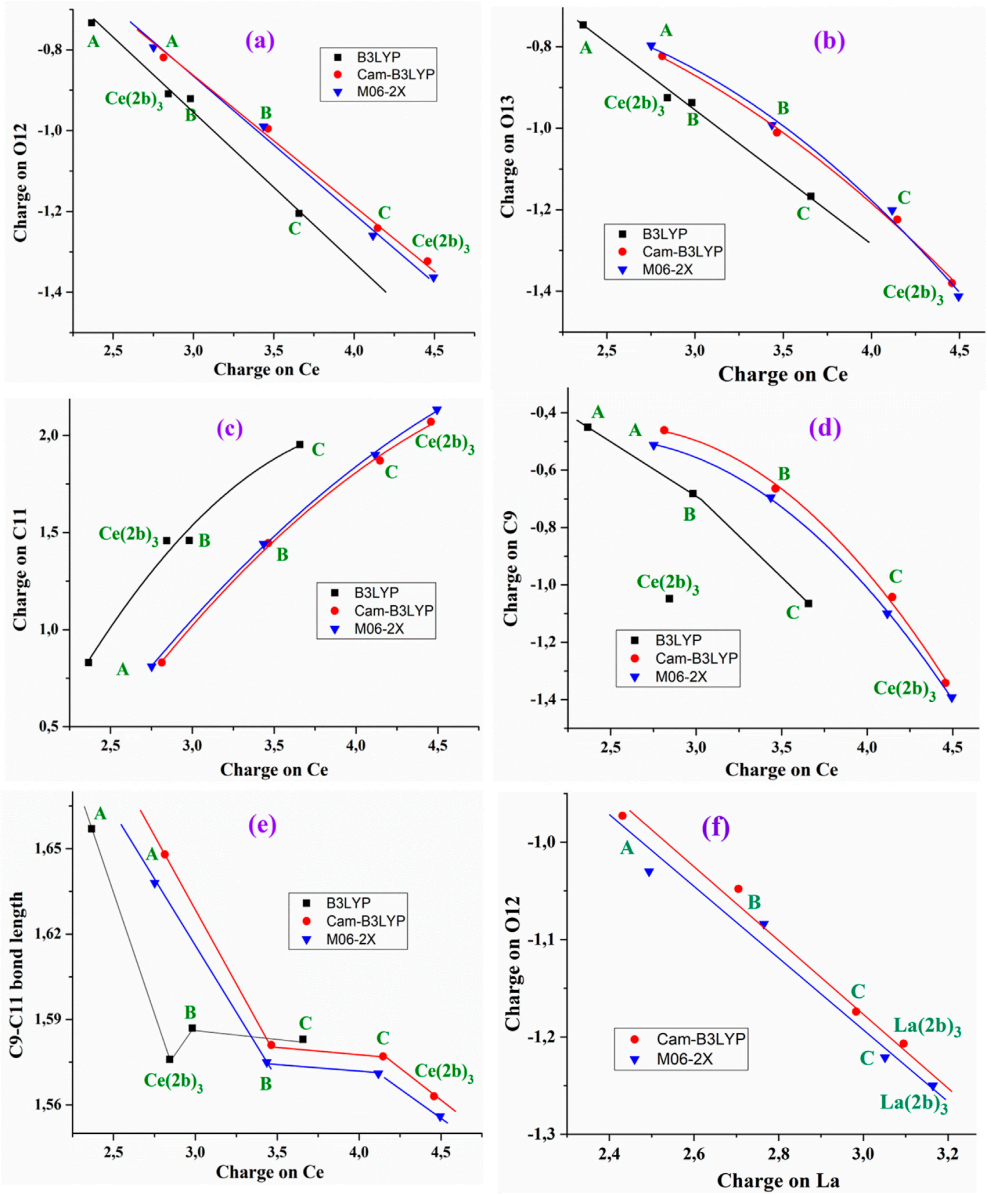
Relationships established at the B3LYP/Cep-4g, CAM-B3LYP/Cep-4g, and M06-2X/Cep-4g levels between the positive calculated atomic APT charge on the cerium ion in the A, B, C, and Ce (2b′)_3_ complexes with **(A)** the atomic charge on O_12_, **(B)** the atomic charge on O_13_, **(C)** the atomic charge on C11, **(D)** the atomic charge on C9, **(E)** the C9–C11 bond length, and **(F)** relationship established at the CAM-B3LYP/Lanl2dz and M06-2X/Lanl2dz levels between the atomic APT charge on the lanthanum ion and the atomic charge on O_12_.

The charge of the Ce(III) ion has a large influence on the bond lengths and atomic charges in the A-, B-, C-, and Ce (2b′)_3_ complexes, and therefore, several relationships can be well-established by the B3LYP, CAM-B3LYP, and M06-2X methods, as shown in Figure 2. For instance, an increase in the positive calculated atomic APT charge on the cerium atom shows good linear relationship to an incremental increase in the negative charge on O_12_ and O_13_ atoms, as shown in Figures 2A,B. The magnitude of the electronic charge lost by the Ce(III) ion is almost the same as that transferred to both O_12_ and O_13_. Increasing the ligand size appears to facilitate this negative electron transfer. Calculations show a large change in the A complex when the triazole ring is inserted (B-complex) and a notable increase with the additional insertion of the aryl ring (C complex). The addition of the pyrrolidine ring increases that electron transfer only a little. B3LYP differs in this point. The values by CAM-B3LYP and M06-2X methods are very close, deviating somewhat from those calculated by the B3LYP method. These increased deviations are presented in Figures 2C–E. Moreover, B3LYP shows large discrepancies in the calculated values for the Ce (2b′)_3_ complex, and for this reason, the results obtained by B3LYP were not discussed in the present study.

The increase in the negative charge on the oxygen atoms leads to a similar increase in the positive charge on the C_11_ atom, as shown in Figure 2C. Consequently, the atomic charge on C_9_ is negative and increased, as shown in Figure 2D. These features show the facility for electron transfer in the presented complexes.

The charge variation in the cerium ion was also related to the C_9_–C_11_ bond length, as shown in Figure 2E. Although this relation is not linear, there is a relationship between both parameters. In general, the charge variation in the ligand atoms leads to changes in the geometrical parameters. Supplementary Figure S3 shows several relationships with the increase in the cerium charge, leading to an increase in the Ce–O_12_ and C=O_12_ bond lengths and a decrease in Ce–O_13_. The relationships plotted in Figure 2 have also been observed with the lanthanum ion (Alcolea Palafox et al., 2023a). For example, the relationship calculated with the better Lanl2dz basis set between the atomic APT charge on the lanthanum ion and the atomic charge on O_12_ is shown in Figure 2F. A larger increase is observed in the positive charge value on the central cerium cation and atom C_11_ with the increase of the negative charge in C_9_, O_12_, and O_13_ in comparison with La cations.

### 3.3 Molecular properties

Several parameters, such as rotational constants, heat capacity at constant volume, dipole moments, molecular orbitals, and other global chemical descriptors in the A, B, C, and Ce (2b′)_3_ complexes, have been determined and are presented in Table 4. The large symmetry obtained in the optimized complexes leads to values of the rotational constant similar in A, B, and C. Very small differences are observed in its calculation by the four DFT methods used. Its value decreases with the increase in the systems’ complexity, i.e., from A to Ce (2b′)_3_.

**TABLE 4:** Molecular properties and global chemical reactivity descriptors (eV) calculated at different DFT levels in the Ce(III) complexes.

Molecular property	A complex		B complex		C complex		Ce(2b′)_3_			
	CAM-B3LYP	M06-2X	CAM-B3LYP	M06-2X	CAM-B3LYP	M06-2X	B3LYP	D3-B3LYP	CAM-B3LYP	M06-2X
Rotational constants (GHz): A	0.575	0.582	0.133	0.135	0.023	0.028	0.018	0.018	0.018	0.019
B	0.572	0.582	0.129	0.131	0.023	0.020	0.018	0.018	0.018	0.017
C	0.328	0.331	0.070	0.071	0.013	0.012	0.011	0.011	0.011	0.011
C_v_ (cal/mol·K)	51.7 134.3	53.6 142.9	86.8 188.4	83.8 181.6	163.8 297.1	162.4 296.2	231.5 387.8	231.0 388.5	225.9 383.0	224.1 379.4
S (cal/mol·K)										
Dipole moment (debye)	0.098	0.130	0.040	0.036	5.416	2.571	11.523	10.916	10.604	5.193
HOMO	−0.553	−0.565	−0.512	−0.513	−0.423	−0.421	−0.342	−0.344	−0.390	−0.390
LUMO	−0.290	−0.350	−0.288	−0.346	−0.279	−0.337	−0.306	−0.305	−0.239	−0.296
E_g_	0.263	0.214	0.224	0.167	0.144	0.083	0.107	0.039	0.151	0.093
IP	0.553	0.565	0.512	0.513	0.423	0.421	0.342	0.344	0.390	0.390
EA	0.290	0.350	0.288	0.346	0.279	0.337	0.306	0.305	0.239	0.296
χ	0.421	0.457	0.400	0.430	0.351	0.379	0.324	0.324	0.315	0.343
η	0.131	0.107	0.112	0.083	0.072	0.042	0.018	0.019	0.076	0.047
S	0.066	0.054	0.056	0.042	0.036	0.021	0.009	0.009	0.038	0.023

The constant volume heat capacity (C_v_) value differs slightly between the DFT methods used, being slightly higher with CAM-B3LYP compared to M06-2X, with the exception of the A complex. As expected, its value markedly increases with system complexity. Entropy (S) values also increase with the system complexity, although to a lesser extent.

Dipole moment values indicate an almost null water solubility in A and B complexes but a remarkable increase when the aryl and pyrrolidine rings are inserted in the ligands, complexes C and Ce (2b′)_3_. In these C and Ce (2b′)_3_ complexes, the calculated value by M06-2X is almost half that by the three other DFT methods.

**FIGURE 3:**
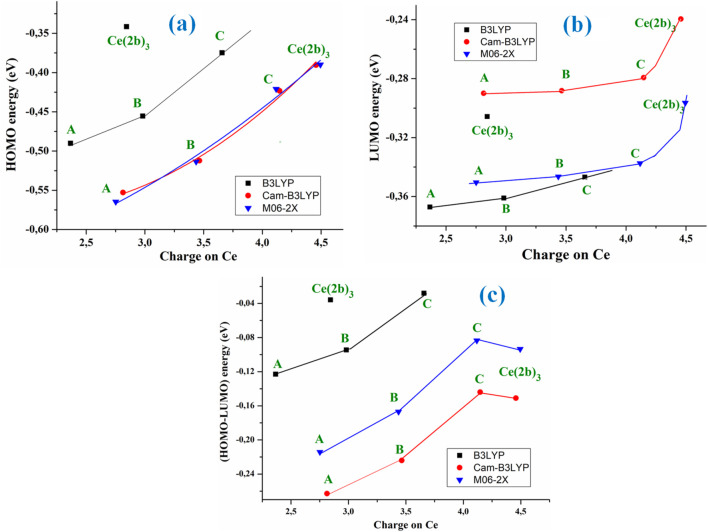
Relationships between the positive calculated atomic APT on the cerium ion in the A, B, C, and Ce (2b′)_3_ complexes using the B3LYP, CAM-B3LYP, and M06-2X methods with **(A)** the HOMO energy orbital, **(B)** the LUMO energy orbital, and **(C)** the (HOMO–LUMO) energy difference.

HOMO (highest occupied molecular orbital) and LUMO (lowest unoccupied molecular orbital) values have also been determined. Their values slightly decrease as system complexity increases, and they appear in a linear relationship with the atomic charge on the Ce ion, with the exception of the Ce (2b′)_3_ complex values, as shown in Figures 3A,B.

The M06-2X and CAM-B3LYP methods calculate almost the same HOMO energy orbital value but differ largely in the LUMO value. With these computed energies, the global chemical reactivity descriptors were determined, which facilitates a better understanding of the stability and reactivity of the four Ce(III) complexes studied in the present work. The energy gap (Eg) between HOMO and LUMO frontier orbitals (HOMO–LUMO gap) appears as one of the meaningful characteristics of molecules, and it facilitates the characterization of chemical reactivity and kinetic stability. By all methods, the Eg value was linearly related to the Ce atomic charge (Figure 3C) and it decreases as the complexity of the system increases, with the exception of the Ce (2b′)_3_ complex that has a similar value to the C complex. A high Eg value shows that the molecule (or complex system) is less polarizable, which is generally related to low chemical reactivity and hence - high kinetic stability. Since the Eg values decrease with the increase in system complexity and the lowest value appears in the C complex, it means that this C complex is more reactive than Ce (2b′)_3_, with the A complex being the most stable one. The pyrrolidine ligand appears to slightly reduce the reactivity of the Ce (2b′)_3_ complex.

The low Eg values obtained in the Ce (2b′)_3_ and C complexes with the four DFT methods used reveal their noticeable chemical reactivity, and, therefore, small energies are required for excitation.

With the HOMO and LUMO energies, several global chemical reactivity descriptors were calculated according to the following well-known formulas (Equations 11–15):

$$\text{IP}=‐\;E_{\text{HOMO}},$$ (11)

$$\text{EA}=‐E_{\text{LUMO}},$$ (12)

$$\chi =‐\;\left(E_{\text{HOMO}}+E_{\text{LUMO}}\right)\;/2,$$ (13)

$$\eta =\left(E_{\text{LUMO}}\;‐\;E_{\text{HOMO}}\right)\;/2,$$ (14)

$$S=½\frac{1}{2}\;\eta .$$ (15)

Due to the large reactivity of the Ce (2b′)_3_ complex, its computed ionization potential (IP) value appears slightly lowered with the four DFT methods used. It is noted that the calculations by CAM-B3LYP and M06-2X methods lead to the same value, 0.390 eV, remaining very close to those determined by B3LYP and D3-B3LYP methods. Compared to the other Ce(III) complexes (A, B and C), these values are the lowest, increasing as complexity decreases (the Ce atomic charge decreases).

Electron affinity (EA) appears to be noticeably lower than IP, and it slightly increases in the complexes as complexity decreases, as expected. The M06-2X method predicts higher values than the CAM-B3LYP method. The low electronegativity (χ) observed is in agreement with neutral complexes, and it slightly increases as complexity decreases. The chemical hardness (η) and global softness (S) values indicate the opposition of a system to an alteration in its number of electrons. These values are low in the investigated complexes, and they slightly increase as complexity decreases. When the values of η are low, the system (or the complex system in our case) is named soft; when they are high, the system is identified as hard. Therefore, according to the low values obtained in our complexes, they are soft systems, with small gaps and an easily modified electron density.

In addition, the significant difference should be noted in the global chemical reactivity descriptors of the complex Ce (2b′)_3_ and the same complex with La^3+^, the La (2b′)_3_. For instance, all level results showed an increase in the dipole moment of the cerium complex. Such a deviation in properties can lead to different biological behavior and both qualitative and quantitative differences in their interaction with biological targets.

### 3.4 Vibrational analysis

The confirmation of the proposed molecular structure of the synthesized complex Ce (2b′)_3_ was achieved through a detailed analysis of the calculated and experimental IR and Raman spectra. For this purpose, a first theoretical–experimental comparison of the full IR spectra in the 3,750–400 cm^−1^ range has been carried out, and it is shown in Supplementary Figure S4 with the scaled values obtained by the four DFT levels used. The same comparison but with the Raman spectra and in the 3,750–0 cm^−1^ range is included in Supplementary Figure S5. The identification and characterization of all vibrations in the 3,500–400 cm^−1^ range is collected in Supplementary Table S1 for the calculations with the CAM-B3LYP method, while the results with the M06-2X method are included in Supplementary Table S2. All values correspond to the most stable conformer, with the arrangement of the ligands shown in Figure 1. Because these tables are too long, a resume is included in Table 5, including only the frequencies with high IR or Raman intensities, as well as those characteristics of the complex. Table 5 is divided into two parts corresponding to the results by the CAM-B3LYP and M06-2X methods.

**TABLE 5:** Calculated, scaled, and experimental wavenumbers (ν, cm^-1^) in the Ce (2b′)_3_ complex by CAM-B3LYP and M06-2X methods. Relative infrared intensity (A) in %, relative Raman intensity (S) in %, and Raman depolarization ratios for plane (DP) and unpolarized incident light (DU). For each vibration of the tetramer, the wavenumber with the highest IR intensity is indicated in bold style and that with the highest Raman intensity is indicated in italic style. The relative IR and Raman intensities were only shown for these wavenumbers. DP and DU values were from the most intense Raman line. The number of the ring mode corresponds to Wilson’s notation (Varsányi, 1974).

Calculated by CAM-B3LYP					scaled		Experiment		Characterization by CAM-B3LYP
ν	A	S	DP	DU	LSE	PSE	IR	Raman	
3324, 3323, 3323	47	1	0.70	0.82	3057	3043	2968.1 s		20b, ν(C5–H) in aryl (100)
3312, 3312, 3312	4	3	0.64	0.78	3048	3034			7b, ν(C6–H) in aryl (100)
3303, 3303, 3303	13	0	0.75	0.86	3040	3028			20a, ν(C2–H) in aryl (100)
3211, 3211, 3211	5	3	0.01	0.02	2960	2957	2872.1 m		ν_s_ (C–H) in C15H_2_ in pyrrolidine (100)
***1664***, 1660, 1660	56	35	0.09	0.16	1623	1671	1577.2 br, vs		ν(C_8_–N_14_) (45) + ν_s_ (CC) (34)
1644, 1644, 1644	0	0	0.22	0.36	1605	1653			8b, ν(C=C) in aryl (89)
1635, 1635, 1635	0	92	0.18	0.31	1598	1645		1595.0 vs	8a, ν(C=C) in aryl (82)
1558, **1525**, *1525*	97	100	0.75	0.86	1503	1546	1500.1 s	1504.5 s	ν_as_ (COO) + ν(C_9_-C_11_) + ν(C_4_N) + 19a,ν(CC,CH)
*1475*, **1464**, 1464	100	60	0.04	0.08	1450	1491	1484.4 vs		19a,ν(CC,CH) + ν(C_9_-C_11_) + ν(triazole)
*1385*, 1383, 1383	7	7	0.04	0.07	1380	1417	1418.0 w		ν_as_ (NNN, CN) + 19a, ν(CC)(35) + ν_s_ (COO)
1362, 1362, 1362	2	1	0.75	0.86	1362	1398	1398.3 m		19b, ν(CC)(85)
*1347,* **1339**, 1339	26	12	0.10	0.18	1342	1377	1372.2 vs	1376.8 vs	ν_s_ (COO) + ν(triazole) + 3,δ(CH) + δ(pyrrolidine)
1337, 1336, 1336	7	16	0.05	0.09	1339	1374			ν_s_ (COO) (49) + δ(pyrrolidine) + ν(triazole)
1306, 1305, 1305	6	1	0.75	0.86	1312	1345	1343.1 s		δ_s_ (C–H) in pyrrolidine +ν_as_ (COO)
1303, 1301, *1301*	3	19	0.75	0.86	1309	1342			ν_as_ (CO_12_) + 3, δ(C-H) aryl + δ(triazole)
*1259*, **1248**, 1248	40	3	0.35	0.52	1263	1293	1301.8 vs		ν(triazole) + ν_as_ (CO_13_) + Γ(pyrrolidine)
1238, *1234*, **1233**	41	7	0.75	0.86	1250	1279	1285.1 s		ν_as_ (CO_13_) + ν(C-N) triazole + Γ(pyrrolidine)
1216, 1215, 1215	3	3	0.75	0.86	1234	1262			ν(CO_12_) + ν(C–N) triazole + δ(C–H) in aryl
1189, 1188, 1188	1	0	0.75	0.86	1211	1237	1246.8 m		δ_s_ (C–H) in pyrrolidine
1163, 1163, 1163	1	0	0.75	0.86	1190	1214	1218.1 m		γ_as_ (C–H) in pyrrolidine
1131, 1130, 1130	3	1	0.75	0.86	1161	1183	1178.3 m		γ_as_ (C–H) in pyrrolidine + ν_s_ (NNN)
1112, 1109, ***1109***	9	2	0.75	0.86	1143	1164		1171.5 w	ν_s_ (COO) + ν(NCCN) + γ_s_ (CC,CH)
1011, 1010, 1010	4	1	0.75	0.86	1057	1071	1091.2 vs	1089.9 m	ν(triazole) + ν(C–CL) + ν_s_ (COO) + 18a, δ(CC,CH)
956, 956, 956	0	0	0.73	0.84	1011	1020	1011.9 m	1012.8 w	γ_as_ (CC,CH) in pyrrolidine
932, 931, 931	1	0	0.09	0.16	989	996	969.1 vs		ν_as_ (triazole) + ν_s_ (COO) + γ(CC,CH)
887, 887, 887	0	4	0.02	0.04	951	954			γ(CC) pyrrolidine + ν_as_ (NNN)
840, 839, 839	4	22	0.04	0.08	909	908	914.3 w	970.2 s	ν_as_ (NNN) + 12, δ(CCC) in aryl
742, 742, 742	9	0	0.75	0.86	826	815	857 w-m		17b, γ(C–H) in aryl
*713*, **709**, 709	4	11	0.07	0.13	797	783	829.9 vs		γ_s_ (COO) + ν(triazole) + γ(CC) pyrrolidine
567, ***557***, 557	12	2	0.75	0.86	666	636	654 m		γ(triazole) + δ_as_ (COO) + 4, γ(CCC)
555, 554, 554	4	1	0.75	0.86	663	633	647.1 m		Γ(triazole) + δ_as_ (COO) + 4, γCCC)
474, 474, 473	16	2	0.05	0.10	594	554	509.0 m		δ_as_ (COO) + δ(triazole) + δ(CC) in aryl
371, ***371***, 369	4	0	0.75	0.85	505	452	466.9 m		ν(aryl, C–CL) + δ(COO) + δ(triazole)

Calculated by M06-2X			Scaled		Experiment		Characterization by M06-2X
ν	A	S	LSE	PSE	IR	Raman	
3326, 3326, 3325	37	1	3050	3036	2968.1 s		7b, ν(C_5_–H) in aryl (100)
3314, 3314, 3313	3	1	3039	3027			20b, ν(C_6_–H) in aryl (100)
3308, 3308, 3307	15	0	3034	3022			20a, ν(C_2_–H) in aryl (100)
3223, 3223, 3223	6	5	2961	2957	2872.1 m		ν_s_ (C–H) in C15H_2_ pyrrolidine (100)
*1702*, **1700**, 1698	46	20	1649	1699	1577.2 br, vs		ν(C_8_-N_14_) + ν_s_ (NNN, NC) in triazole
1652, 1651, 1651	0	1	1607	1655			8b, ν(C=C) in aryl (93)
1646, 1646, 1645	1	100	1603	1651		1595.0 vs	8a, ν(C=C) in aryl (93)
1584, ***1550***, 1549	100	73	1520	1565	1500.1 s	1504.5 s	ν_as_ (COO) +ν(C_9_-C_11_) + ν_s_ (CCN) triazole + ν(C_4_-N_4_)
*1493*, 1482, **1481**	94	98	1461	1503	1484.4 vs		19a, ν(CC, CH) + ν(C_4_–N_4_) + ν(C_9_–C_11_)
*1407*, **1405**, 1405	5	10	1395	1434	1418.0 w		ν_as_ (NNN) in triazole +19a, ν(CC) in aryl
1378, 1378, 1378	2	0	1372	1409	1398.3 m		19b, ν(CC,CH) in aryl
1358, 1357, **1352**	28	9	1349	1386	1372.2 vs	1376.8 vs	ν_s_ (COO) + ν_s_ (C_9_–N_10_) triazole +19b, ν(CC,CH) in aryl
1345, 1344, 1344	8	19	1343	1378			ν_as_ (NNN) + ν_as_ (COO) + 19a,ν(CC) + δ(CC) pyrrolidine
1330, **1325**, *1325*	2	14	1326	1361			ν_as_ (COO)+ν_as_ (triazole) +ν_as_ (CNC) pyrrolidine +19a,ν(CC)
1309, 1308, 1308	0	4	1312	1345	1343.1 s		δ_s_ (C–H) in pyrrolidine + ν_s_ (NNN)
*1283*, 1274, **1269**	81	11	1278	1309	1301.8 vs		ν(triazole) (45)+ν(CO_13_)(40)+ δ_s_ (CC,CH) pyrrolidine (12)
1242, 1242, 1241	1	4	1255	1285	1285.1 s		ν_as_ (CO_12_) + ν_s_ (C_9_NN) triazole + δ_s_ (CC,CH) in pyrrolidine
1193, 1192, 1192	1	1	1212	1238	1246.8 m		δ_s_ (C–H) in pyrrolidine
*1168*, **1166**, 1166	5	1	1189	1214	1218.1 m		γ_as_ (C–H) in pyrrolidine +3, δ(CH) + ν_s_ (NNN)
1134, 1132, ***1131***	8	1	1159	1182			ν_s_ (COO) + ν(triazole) + δ_as_ (C–H) in pyrrolidine
1119, 1119, 1118	1	1	1149	1170	1178.3 m	1171.5 w	γ_as_ (C–H) in pyrrolidine + ν_s_ (triazole)
1027, 1025, ***1025***	4	1	1068	1083	1091.2 vs	1089.9 m	ν_s_ (NNN) + ν(C–CL) + 18a, δ(C–H) in aryl
959, 958, 958	0	1	1010	1019	1011.9 m	1012.8 w	γ_as_ (CC, CH) in pyrrolidine
945, 944, 944	3	1	998	1006			ν_as_ (NC_9_–C_8_) + δ_as_ (CC, CH) in pyrrolidine
921, 920, 920	3	1	977	983	969.1 vs		ν_as_ (NNN) + 18a, δ(C–H) in aryl
851, 851, 851	2	19	918	918		970.2 s	ν_as_ (NNN)(36) + 12, δ(CCC) in aryl
751, 750, 750	7	0	831	821	857 w-m		17b, γ (C–H) in aryl
*725*, 724, **723**	3	10	808	795	829.9 vs		γ_s_ (COO) + γ(CC, CH) pyrrolidine +δ(triazole)
***575***, 573, 573	0	1	680	652	654 m		γ(triazole) + γ_as_ (COO) + 8b, δ(CCC) in aryl
572, 565, ***564***	11	2	671	641	647.1 m		Γ(triazole) + γ_as_ (COO)
*478*, 476, **475**	16	2	594	554	509.0 m		δ_as_ (COO) + δ(triazole) + δ(CC) in aryl
372, 370, ***363***	4	1	498	443	466.9 m		ν(aryl, C–CL) + δ_as_ (COO) + δ(triazole)

#### 3.4.1 General comparison of the IR and Raman spectra

In the first comparison of the scaled IR spectra with the experimental spectra plotted in Supplementary Figure S4, the following observations are pointed:

(i) A good agreement between the scaled theoretical spectra with the experimental spectra has been noted. In particular, the scaled strongest vibrations have their corresponding vibrations in the experimental spectrum. This feature confirms the scaling carried out on the calculated wavenumbers; therefore, the applied theoretical methods appear appropriate. Thus, in general, the assignments proposed could be considered true, identifying most of the computed modes in their normal ranges.
(ii) A very broad and strong band centered at 3400.8 cm^−1^ has been observed in the experimental spectrum. Judging by its position and broad intensity, it can be only assigned to the O–H stretching ν mode corresponding to water molecules strongly H-bonded to the nitrogen and oxygen atoms of the three 2b′ ligands in the Ce (2b′)_3_ complex. These water molecules were not included in our optimized theoretical complex due to the fact that in previous studies with La(III) complexes, the water molecules only slightly affected the carboxylate group, the other groups remaining unaffected (Peica et al., 2006). This hydration appears due to the spatial arrangement of the ligands in the complex, which leaves cavities that can be occupied by water molecules. As expected, this band is not observed in the Raman spectrum.
(iii) Another broad but very strong experimental IR band is observed at 1577.2 cm^−1^. Its large broadening can be interpreted as a result of the additional contribution of the in-plane bending δ(OH) mode of these hydrated water molecules to the main assignment of this band corresponding to the C_8_–N_14_ and C–C stretching, as shown in Table 5.
(iv) A noticeable resemblance between the scaled spectra obtained by the D3-B3LYP, CAM-B3LYP, and M06-2X methods that include long-range correction has been observed, while that by B3LYP differs noticeably. As compared to the experimental spectrum, the two best methods are CAM-B3LYP and M06-2X, and for this reason, their spectra were analyzed in detail and are included in Table 5. In this analysis, the scaled wavenumbers by CAM-B3LYP appear slightly more accurate than by M06-2X. This is why CAM-B3LYP has been mainly utilized in the experimental spectra assignment.
(v) The coordination of the 2b′ ligands to the Ce(III) ion remarkably modified the IR and Raman spectra. The spectra appear to differ from those obtained with the 2b′ ligand anion alone (Alcolea Palafox et al., 2023a).

The same analysis has been carried out using the Raman spectra, as shown in Supplementary Figure S5. In this case, the scaled spectra show large differences among the DFT methods. CAM-B3LYP and M06-2X methods again seem to be more accurate with bands showing good agreement with the experimental bands. Unfortunately, the experimental spectrum appears with noticeable background noise, which complicates the detection and further analysis of all weak lines, making this comparison difficult. The appearance of a broad Raman line with medium intensity at 73.1 cm^−1^ was observed in the experimental spectrum, which has not been detected in the theoretical spectra. Due to this feature, it was not shown in Table 5.

#### 3.4.2 Specific comparison of the IR and Raman spectra

**FIGURE 4:**
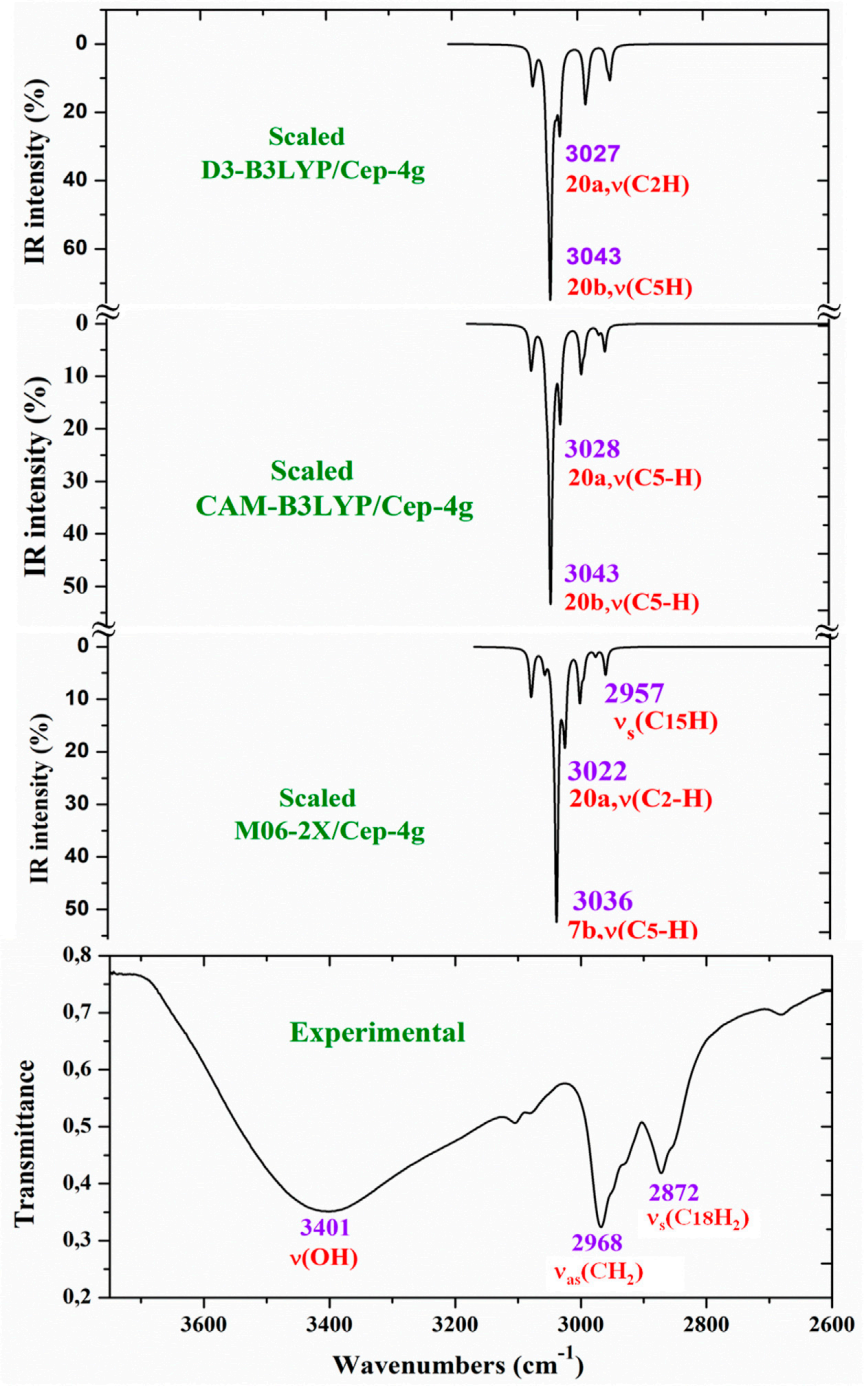
Comparison of the scaled IR spectra with the B3LYP, CAM-B3LYP, and M06-2X methods by the PSE procedure with the experimental spectrum in the 3750–2600 cm^−1^ range.

**FIGURE 5:**
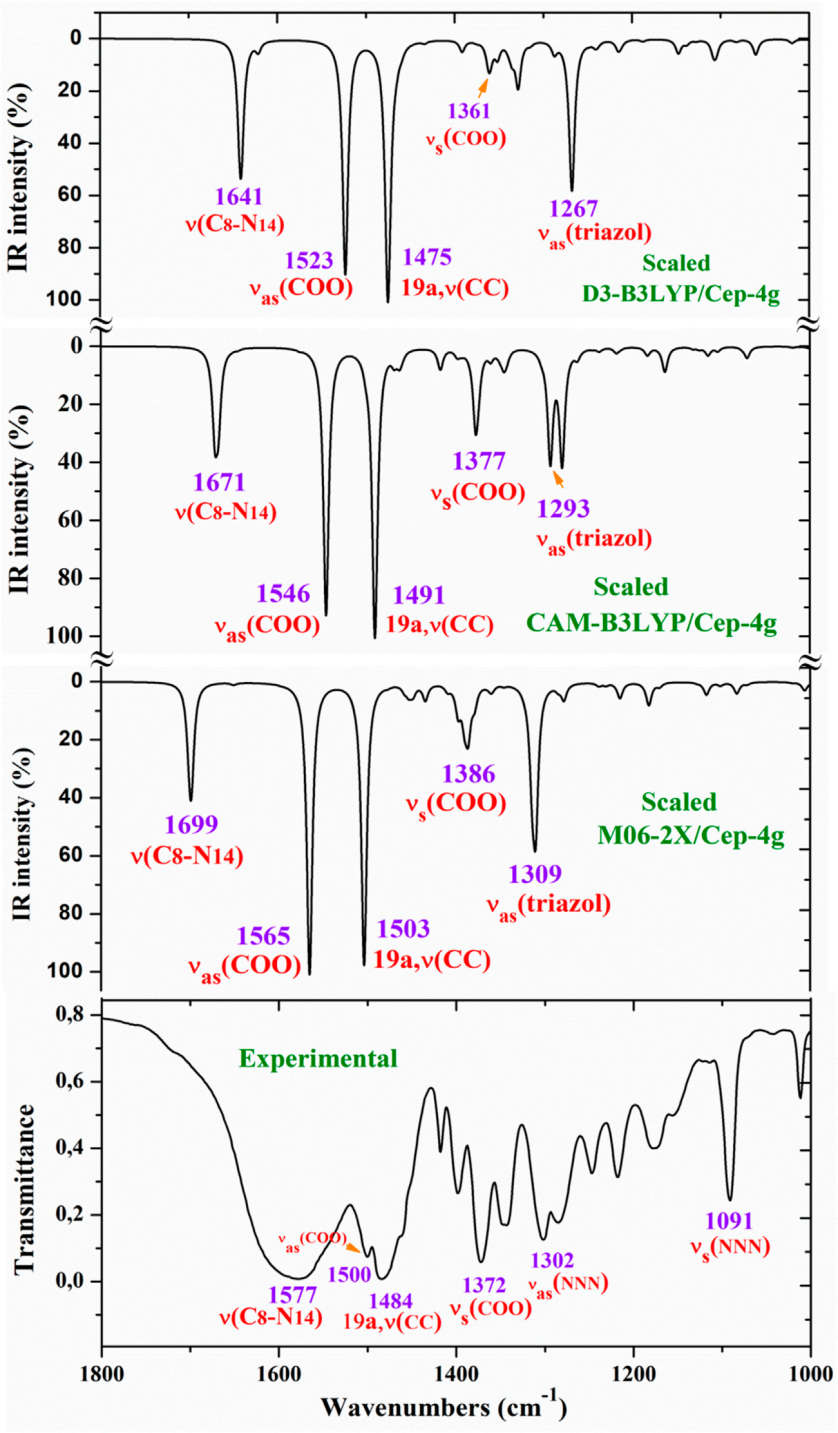
Comparison of the scaled IR spectra at three different levels by the PSE procedure with the experimental spectrum in the 1800–1000-cm^−1^ range.

**FIGURE 6:**
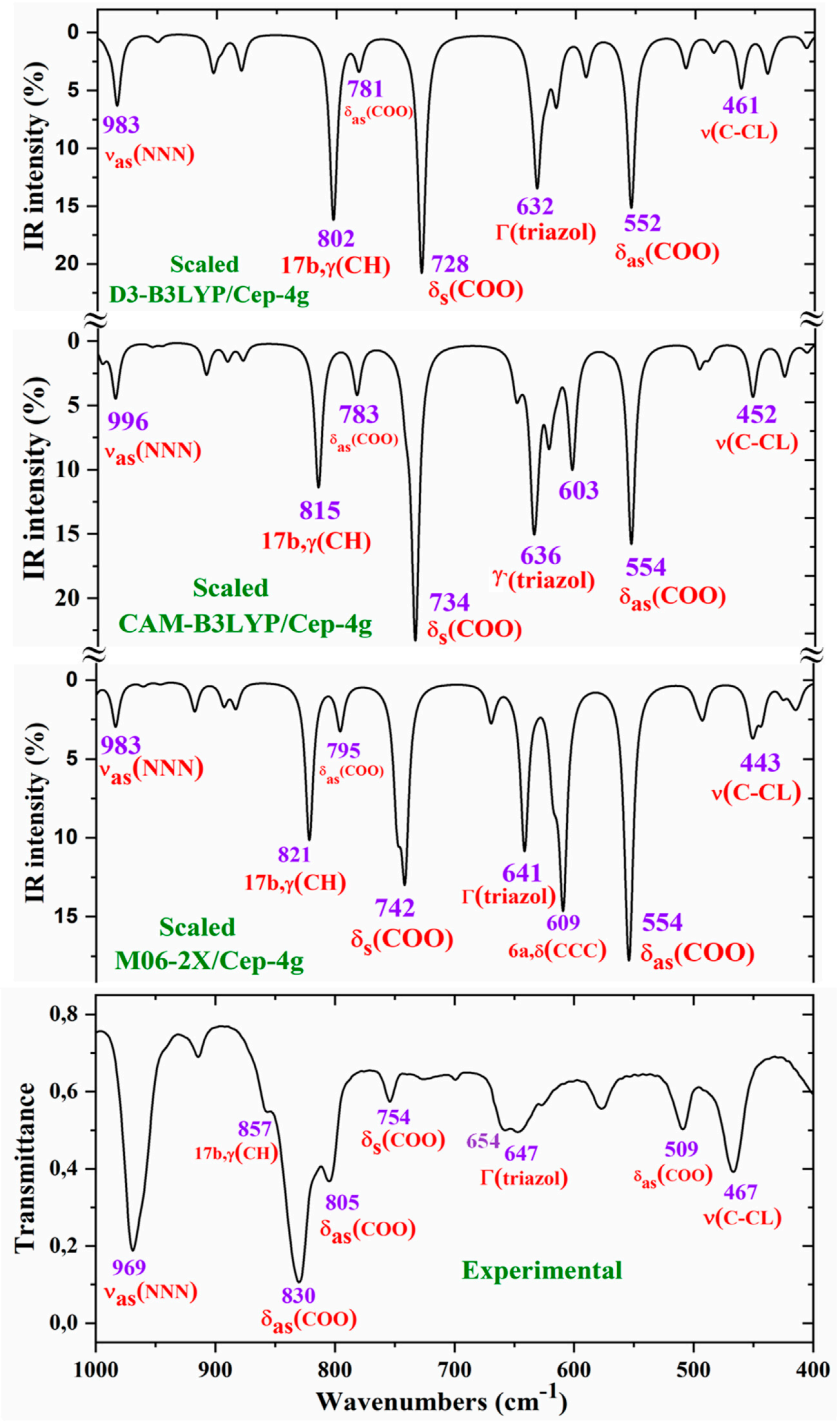
Comparison of the scaled IR spectra at three different levels by the PSE procedure with the experimental spectrum in the 1000–400-cm^−1^ range.

**FIGURE 7:**
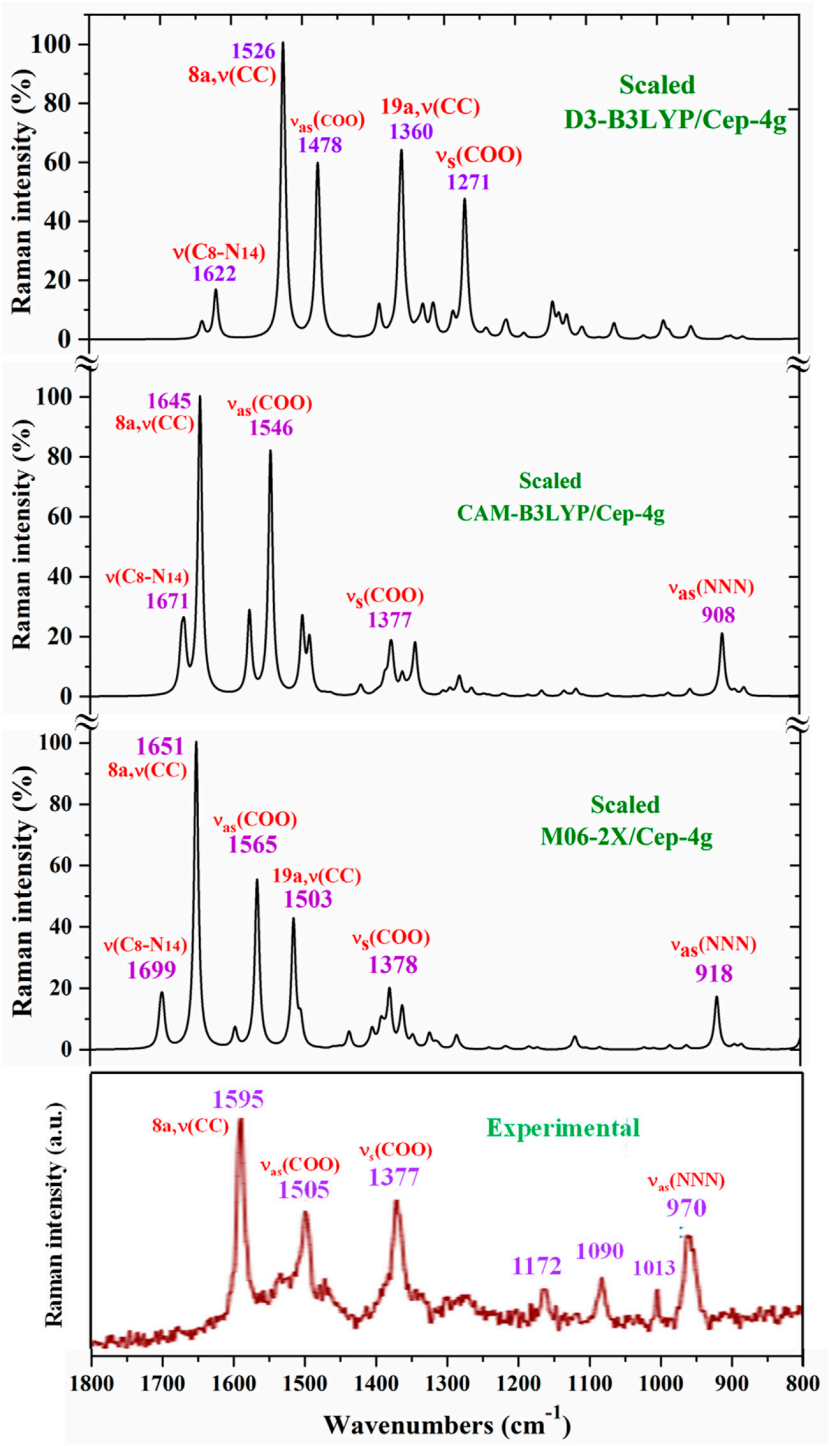
Comparison in the 1800–800-cm^−1^ range of the scaled Raman spectrum by the PSE procedure and at three different levels with the experimental spectrum.

For a comprehensive and specific comparison of the distinct scaled and experimental frequencies, the spectra were divided into three ranges. In the IR spectra, these ranges were 3750–2600 cm^−1^ (Figure 4), 1800–1000 cm^−1^ (Figure 5), and 1000–400 cm^−1^ (Figure 6). In the Raman spectrum, the comparison was only carried out in the 1800–800-cm^−1^ range, as shown in Figure 7, in which the Raman lines can be clearly identified, because of the noticeable background noise of the experimental spectrum. In these figures, for simplicity, only the assignment of strong and characteristic vibrational modes was included. For the final assignment of the experimental bands, characterization obtained by the different DFT methods was considered, as well as the assignment reported in related molecules.

For this purpose, a resume with the most characteristic frequencies determined in the Ce (2b′)_3_ complex with the CAM-B3LYP and M06-2X methods is shown in Table 5. The first column presents the three computed frequencies for each mode related to the three 2b′ ligands of the complex. Of these three frequencies, the frequency with higher computed IR intensity was typed in bold style, whereas the frequency with higher Raman intensity was typed in italic style. This notation was not used if the intensity of these three wavenumbers is similar or very weak. The second and third columns list the relative IR and Raman intensities (%) corresponding to the frequencies of the first column typed in bold style and in italic style, respectively. These relative intensities were determined by normalizing each computed data to the strongest intensity of the spectrum. In the CAM-B3LYP method was included two new columns, the fourth and fifth, with the Raman depolarization ratios for plane (DP) and unpolarized incident light (DU), respectively.

The values included correspond to the wavenumbers typed in italic style in the first column. The scaled wavenumbers by the LSE or PSE procedures were listed in the next two columns. For simplicity, only the wavenumbers typed in bold style are included as scaled values in these two columns. The observed experimental IR and Raman bands with their corresponding intensities were collected in the next two columns, respectively. Finally, the last column shows the principal characterization of the computed wavenumbers determined by the CAM-B3LYP (first part of the Table) and M06-2X (second part of the Table) methods. The % contribution (PEDs) of the distinct modes to a calculated wavenumber was added only in few cases.

The main purpose of this vibrational study is the identification and characterization of the synthesized Ce (2b′)_3_ coordination complex. For this reason, the main focus was on the most characteristic modes and the strongest IR and Raman bands, in order to validate the structure that was planned and optimized in Figure 1. Since the spectra with the CAM-B3LYP method seem to be the most precise, their frequencies have been mainly used for discussion. In specific cases, the scaled values obtained by the M06-2X method were also discussed. For simplicity, the scaled values obtained through the PSE scaling procedure were mainly used in the discussion due to their slightly better agreement with experimental results than those with the LSE procedure.

The analysis and assignment of different vibrational modes have been carried out under the following sections: 1) the COO^−^ group modes, 2) the triazole ring modes, and 3) the aryl ring modes. Due to the low quality of the Raman spectra, the vibrational modes with the Ce(III) ion were not detected in the experimental spectrum, and therefore, they were not discussed in the present work.

##### 3.4.2.1 The carboxylate COO^−^ group modes

The vibrations of this group appear to be of great interest because it has been found (Palafox et al., 2023) that the ligand is fixed to the amino acid chain of the receptor through this group. In solid state samples (Sokrates, 2004), the asymmetric ν_as_ (COO^−^) mode of the carboxylate group appears as a strong IR band in the 1600–1560 cm^−1^ range, while the ν_s_ (COO^−^) mode appears at lower wavenumbers, although the spectrum of this region is complicated due to intermolecular H-bonding. In the 2b ligand alone, ν_as_ (COO^−^) stretching has been scaled at 1713 cm^−1^ with very strong IR intensity (Palafox et al., 2023). Nevertheless, in the Ce (2b′)_3_ complex, a noticeable red shift to lower values is expected due to the remarkably longer C-O bonds used to form the six O–Ce coordination bonds. In the CAM-B3LYP method, a large contribution of the asymmetric ν_as_ (COO^−^) mode was identified in the calculated wavenumber at 1525 cm^−1^ (scaled at 1503 cm^−1^via the LSE procedure) with very strong IR intensity, the second strongest of the spectrum, in very good agreement with the experimentally strong IR band at 1500.1 cm^−1^ and with the strong Raman line at 1504.5 cm^−1^. This feature was also observed with the M06-2X method, appearing scaled at 1520 cm^−1^ by LSE, which is in good agreement with the experimental spectrum. Large contributions of the asymmetric stretching vibrations ν_as_ (CO_12_) and ν_as_ (CO_13_) were also identified in the scaled wavenumbers by CAM-B3LYP at 1342, 1293, 1279, and 1262 cm^−1^, which are in good agreement with the experimentally very strong IR band at 1301.8 cm^−1^ and with the strong band at 1285.1 cm^−1^. The M06-2X method confirms these assignments in the scaled values at 1309 and 1285 cm^−1^, respectively.

The largest contribution of the symmetric ν_s_ (COO^−^) mode was found in the calculated wavenumber at 1336 cm^-1^ (scaled at 1374 cm^−1^ via the PSE procedure), which is consistent with that found in the 1420–1400-cm^−1^ range in solid-state samples of related compounds (Sokrates, 2004). A large contribution of the asymmetric stretching vibration ν_s_ (COO^−^) was clearly identified by CAM-B3LYP in the scaled wavenumber by PSE at 1377 cm^−1^ with strong IR intensity and medium Raman intensity, which is in excellent agreement with the very strong bands at 1372.3 (IR) and 1376.8 cm^−1^ (Raman). The M06-2X method confirms this assignment with scaled wavenumbers at 1386 and 1378 cm^−1^.

##### 3.4.2.2 Triazole ring modes

The triazole group vibrations are also of great interest as their nitrogen atoms appear to be weakly H-bonded to the amino acids of the receptor (Palafox et al., 2023). The geometry and vibrational frequencies of the 1,2,3-triazole ring in different triazoles have already been studied (Aziz et al., 2014; El-Azhary et al., 1998; Törnkvist et al., 1991), and our computations and experimental results are in good agreement with them. Therefore, for simplicity, the analysis of different vibrations of this group has only been focused in the assignment of the strongest bands.

*NNN modes:* The ν_as_ (NNN) stretching was characterized by CAM-B3LYP as strongly coupled with the 19a aryl mode in the scaled frequency with weak IR intensity at 1417 cm^−1^, which is in good agreement with the weak experimental IR band at 1418.0 cm^−1^. The results by M06-2X are in good agreement with this assignment with a scaled wavenumber at 1434 cm^−1^ and also with weak IR intensity. Due to the large background noise of the experimental Raman spectrum, the weak lines predicted for this mode were not clearly identified.

Other triazole ring stretching modes with asymmetric character and strongly coupled with other modes were identified in the scaled wavenumbers at 996 and 908 cm^−1^ and are related to the very strong experimental IR band at 969.1 cm^−1^ and weak band at 914.3 cm^−1^, respectively. In the experimental Raman spectrum, this mode appears as a strong line at 970.2 cm^−1^ in accordance with the medium intensity vibration predicted by M06-2X and CAM-B3LYP methods. D3-B3LYP dramatically fails in its prediction, as well as in other bands, and for this reason, their values were not considered. The Raman line detected at 1012.8 cm^−1^ was also assigned to this mode.

The symmetric stretching ν_s_ (NNN) mode was identified at lower wavenumbers and strongly coupled with the symmetric ν_s_ (COO^−^) mode, as well as with other ring modes. It was predicted by CAM-B3LYP with medium–strong IR intensity at 1377 cm^-1^ and corresponds to the very strong IR band at 1372.2 cm^−1^. The very strong experimental Raman line observed at 1376.8 cm^−1^ can be assigned to this mode or to the neighbor scaled wavenumber at 1374 cm^-1^. A similar scaled wavenumber at 1378 cm^-1^ was calculated by the M06-2X method. In the 2b ligand alone (isolated state), it has been scaled at 1360 cm^-1^ and assigned to the experimental IR band at 1340.5 cm^−1^ (Palafox and Rastogi, 2013).

*C*_*8*_ *–N*_*14*_ *modes:* The stretching mode has been scaled via the LSE procedure with CAM-B3LYP at 1623 cm^-1^ with strong IR intensity, and it corresponds to the very strong and broad band observed in the experimental IR spectrum at 1577.2 cm^−1^. This difference in the wavenumbers and the broadening of the experimental IR band can be due to the contribution of the δ (O-H) mode corresponding to the hydrated water molecules. This assignment was in agreement to that found by M06-2X in the scaled wavenumber at 1649 cm^-1^ by the LSE procedure. This stretching mode was identified in the isolated 2b ligand in the scaled wavenumber at 1556 cm^-1^ (Palafox et al., 2023), which is in good agreement with the experimental bands with medium intensities at 1543.9 cm^-1^ (IR) and 1550.6 cm^−1^ (Raman).

##### 3.4.2.3 Aryl ring modes

The aryl and the pyrrolidine groups appear to provide liposolubility to the complex, facilitating its membrane cell crossing, and in addition, the aryl group establishes π–π and π–alkyl interactions, stabilizing the ligand binding to the amino acids of the receptor (Palafox et al., 2023). For this reason, the vibrations of this aryl group are analyzed in the present study. For the assignments of the ring modes, the Varsanyi notation (Varsányi, 1974) for a 1,4-disubstituted benzene derivative was followed. Therefore, the substituent modes of the aryl ring for stretching vibrations correspond to **7a** and **13** modes, for in-plane vibrations to **9b** and **15** modes, and for out-of-plane vibrations to **10b** and **11** modes. In particular, the C4–N4 bond is represented by **13**, **15**, and **10b** modes, while the C–CL bond is represented by **7a**, **9b**, and **11** modes. In a chloro-substituted benzene derivative (Palafox and Rastogi, 2013), mode **7a** ν(C–CL) was found at 359 cm^−1^, mode **9b** δ(C–CL) at 310 cm^−1^, and mode **11** γ(C–CL) at 93 cm^−1^.

The assignments of many of the remaining ring modes are shown in Table 1S and do not need further analysis; thus, interest was directed only to the strongest vibrations in order to confirm the structure of the synthesized complex.

The aromatic C–H stretching modes appear mainly in the 3200–2950 cm^−1^ region theoretically as almost pure modes (100% PED) with weak or very weak IR and Raman intensities. Thus, only mode **20b** with a scaled value by the PSE procedure at 3043 cm^−1^ correlated with the experimental IR band with strong intensity at 2968.1 cm^−1^. This wavenumber is exactly the same as the wavenumber found in an isolated 2b ligand (Palafox et al., 2023), which can be explained by the negligible effect of the Ce(III) ion on the far aryl ring.

The aromatic C–C stretching vibrations, modes **8a** and **8b**, appear as nearly pure modes but with %PED of approximately 90%. Through the CAM-B3LYP method, mode **8a** was scaled by the LSE procedure at 1598 cm^−1^ with practically null IR intensity, but being the second vibration with the highest Raman intensity, which is in excellent agreement with the very strong Raman line at 1595.0 cm^−1^. A similar scaled wavenumber at 1603 cm^−1^ is predicted by M06-2X. Mode **8b** was calculated by both DFT methods with practically null IR and Raman intensities, and therefore, it has not been detected in the experimental spectra.

Mode **19a** was calculated by both DFT methods with very strong IR and Raman intensities at 1491 cm^−1^, which is in excellent agreement with the experimental strong IR band at 1484.4 cm^−1^. Mode **19b** was scaled at 1398 cm^−1^ with weak IR and very weak Raman intensities, which is in agreement with the experimental IR band with medium intensity detected at 1398.3 cm^−1^.

### 3.5 Free radical-scavenging activity

Oxidative stress (OS) is a physiological state associated with an imbalance between the production and elimination of reactive species in a living organism. In terms of human health, OS is generally associated with the development of a number of pathologies (Hajam et al., 2022). However, there are some cases in which OS is beneficial, particularly in cancer treatment (Van Loenhout et al., 2020), immunological response (Lauridsen, 2019), and/or pharmacological treatment (Albesa et al., 2004) of microbial diseases. Lanthanide ions and their coordination compounds are known for their antimicrobial (Cota et al., 2019; Wakabayashi et al., 2016) and anticancer properties (Patyal et al., 2023), which are associated with their luminescent properties (Zhang et al., 2023) and ionic mimicry, particularly toward iron (Todorov et al., 2019). Herein, the interaction between the novel complex Ce (2b′)_3_ and two hydroxyl radical generating model systems that involve UV-induced water radiolysis (Burns and Sims, 1981) and the physiologically significant Fenton reaction (Levin et al., 2016) is reported. Hydroxyl radicals are highly aggressive reactive species that tend to “attack” double bonds in biomolecules, causing lipid peroxidation and radical chain reactions and, therefore, can ultimately inflict a variety of pathologies in living organisms (Negre-Salvayre et al., 2010). 2,2′-Azino-bis(3-ethylbenzothiazoline-6-sulfonic) free radical (ABTS^·+^) and 2,2-diphenyl-1-picrylhydrazyl radical (DPPH^·^) assays were additionally performed to evaluate the ability of Ce (2b′)_3_ to scavenge radicals by single-electron transfer (SET) and hydrogen atom transfer (HAT) reactions, respectively. The radical-scavenging activities of the sodium salt 2b and its La(III) complex, La (2b′)_3_, have previously been reported (Alcolea Palafox et al., 2023a) and are presented together with the new results for Ce (2b′)_3_ in order to observe the impact (if any) of the type of coordination center on radical-scavenging activity.

#### 3.5.1 Impact of Ce (2b′)_3_ on 2-deoxyribose degradation

**FIGURE 8:**
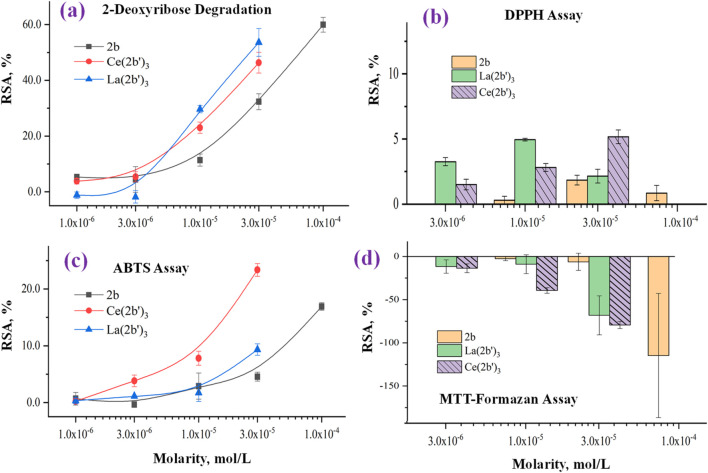
Impact of 2b, Ce (2b′)_3_, and La (2b′)_3_ on **(A)** 2-deoxyribose degradation, **(B)** DPPH^●^, **(C)** DPPH^●^, and **(D)** MTT-formazan transformation by Fenton-generated OH^●^. Data = mean ± StDev, *p* < 0.05, N = 3. A higher result means higher scavenging activity.

The impact of the Ce(III) complex on 2-deoxyribose degradation as a result of UV-induced water radiolysis is presented in Figure 8A. The observed effect with Ce (2b′)_3_ is concentration-dependent, as is the case with the previously reported 2b and La (2b′)_3_. At concentrations below 1·10^−5^ M, the activity of the complex is very mild (RSA<5%). At 1.10^−5^ M and higher, the cerium complex is more active than the ligand in the same concentration. At 3.10^−5^ M, Ce (2b′)_3_ and La (2b′)_3_ have similar activity (RSA = 46 ± 4% and 54 ± 5%, respectively). The observed impact of the coordination center in this model system appeared to be very mild, with both complexes exhibiting similar activity within the tested range of molarities.

#### 3.5.2 Impact of Ce (2b′)_3_ on a model system containing the stable radical DPPH^·^

The ability of Ce (2b′)_3_ to exchange hydrogen with DPPH^·^ is presented in Figure 8B. Previous experiments have shown that the ligand 2b and the complex La (2b′)_3_ manifest very mild activity in this model system - RSA<5% at even the highest concentration with the effect decreasing to 0 at 3·10^−6^ M and lower. The case of Ce (2b′)_3_ is very similar. At 3.10^−5^ M, this complex has RSA = 5.2 ± 0.5%. The effect decreases in a concentration-dependent manner to 1.5% ± 0.4% at 3·10^−6^. DPPH^·^ is scavenged by HAT, which is similar to OH^·^ (Galano, 2015). However, its relatively large size may be implicated in the observed low activity in this model system when compared to the 2-deoxyribose degradation model. Ce (2b′)_3_ is more active than 2b at three times higher molarity, a sign that Ce(III) possibly influences the distribution of electron density within the coordinated ligands, increasing the activity of hydrogen atoms that, in turn, could interact with DPPH^·^.

#### 3.5.3 Impact of Ce (2b′)_3_ on a model system containing the stable radical ABTS^·+^

The ability of the novel complex to participate in electron-exchange reactions with ABTS^·+^ is shown in Figure 8C. A concentration-dependent impact is observed with RSA increasing from 0.24 ± 0.44% at 1·10^−6^ M to 23 ± 1% at 3·10^−5^ M. At the same molarities, the activity of Ce (2b′)_3_ is higher than that of 2b and even the lanthanum(III) counterpart at 3·10^−6^ M or more. The activity of the Ce(III) complex tends to be higher than that of 2b at three times higher concentration. At the highest tested molarity (3·10^−5^ M) Ce (2b′)_3_ has RSA = 23 ± 1%, which is higher compared to 9 ± 1% for La (2b′)_3_ (3·10^−5^ M) and 16.9 ± 0.7% for the ligand 2b (1·10^−4^ M). Unlike La(III), the complexation of 2b with Ce(III) seems to promote electron-exchange with ABTS^·+^ rather than suppress it.

#### 3.5.4 Impact of Ce (2b′)_3_ on MTT-formazan transformation triggered by Fenton reaction-derived hydroxyl radicals

The Fenton reaction is a clinically significant chemical process (Kell, 2009) that involves the transition metal-catalyzed production of hydroxyl radicals from H_2_O_2_. OH^·^ are highly reactive species that tend to attack molecular sites with conjugated double bonds, causing molecular fragmentation, lipid peroxidation, and generation of malondialdehyde-like products. Since both the Fenton reaction and water radiolysis produce OH^·^, similar activity in both model systems would also be expected. This, however, is not the case for Ce (2b′)_3_, which is consistent with previously published observations with 2b and La (2b′)_3_. Results are presented in Figure 8D.

Previous observations demonstrate that in this model system, the ligand 2b is inert at 3·10^−5^ M or less. At 1.10^−4^ M, it actually increases MTT-formazan formation to a significant degree (RSA = −115 ± 72%), a sign of pro-oxidant action. At 3.10^−5^ M, Ce (2b′)_3_ also seems to act as a pro-oxidant (RSA = −79 ± 4%), which is similar to the previously reported La (2b′)_3_ (RSA = − 68 ± 23%). This effect decreases in a concentration-dependent manner. At 1.10^−5^ M, Ce (2b′)_3_ seems to behave as a slightly more potent prooxidant compared to La (2b′)_3_ (RSA = −39 ± 3% and 9.0 ± 10.9% respectively). Ce (2b′)_3_ seems to act as a more potent prooxidant than 2b at concentrations 3·10^−5^ M and lower. At 3.10^−6^ M and 1·10^−5^ M, it increases MTT-formazan transformation to a greater extent than the ligand 2b at three times greater molarity.

#### 3.5.5 Chemiluminometric assays: Impact of Ce (2b′)_3_ on LDCL in the presence of KO_2_ and NaOCl

In previous work Alcolea Palafox et al. (2023a), the effects of 2b and La (2b′)_3_ on LDCL are reported in the presence of NaOCl and KO_2_. The ligand and its La (III) complex behaved as weak pro-oxidants in the presence of superoxide. In the NaOCl model system, the ligand seems to show a tendency to behave as a mild concentration-dependent antioxidant (CL-SI = 64 ± 40%), while the La(III) complex behaved as a pro-oxidant (CL-SI = 186 ± 6%, 3·10^−5^ M). The methodologies involved dissolving the compounds in 100–200 μL of DMSO and further diluting to 10 mL of distilled water. In order to maintain experimental conditions as close to physiological conditions as possible, the experiments were repeated, this time without the application of DMSO, a compound known for its antioxidant activity. The results of the 2b and La (2b′)_3_ experiments (without DMSO), as well as the research on Ce (2b′)_3_, using the updated protocol are shown below.

**FIGURE 9:**
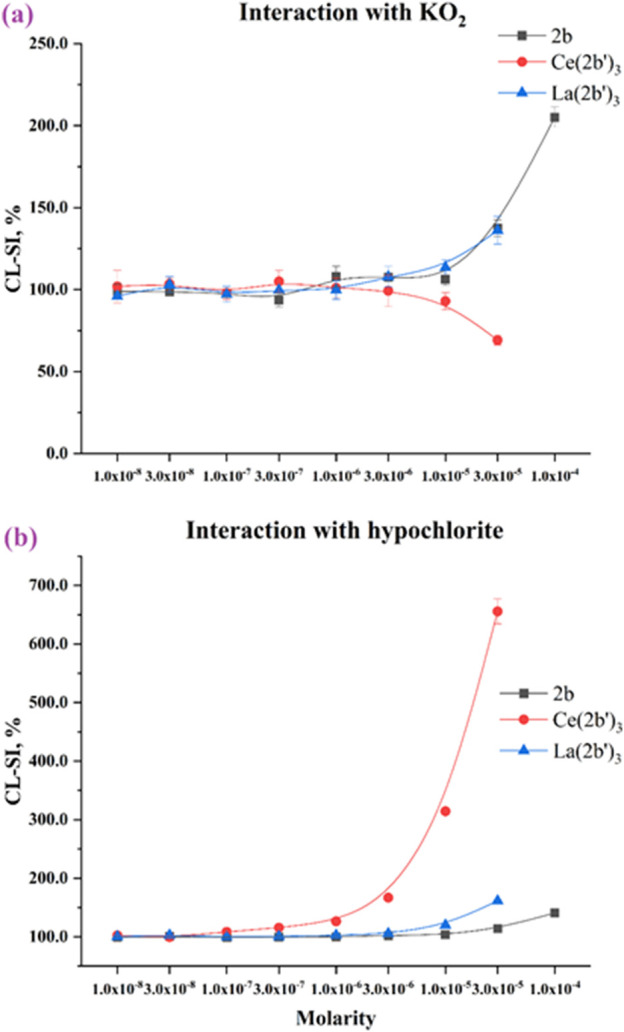
Interaction of the 2b ligand and Ce (2b′)_3_ and La (2b′)_3_ complexes with **(A)** KO_2_ and **(B)** hypochlorite. Data = mean ± StDev, *p* < 0.05, N = 3. A lower result means higher scavenging activity.

The superoxide ion is an oxygen radical that is produced in the human body by one-electron reduction of oxygen. It also participates in the body’s defense against pathogens and in a multitude of cell signaling pathways (Che et al., 2016; Trist et al., 2021). The ability of the ligand 2b, La (2b′)_3_, and Ce (2b′)_3_ to scavenge KO_2_-derived superoxide is presented in Figure 9A.

All three compounds are almost inert at 3·10^−6^ M or lower. As molarities increase, LDCL in the presence of 2b and La (2b′)_3_ increases up to CL-SI = 205 ± 6% for 2b at 1·10^−4^ M and CL-SI = 136 ± 8% for La (2b′)_3_ at 3·10^−5^ M, a sign of a the pro-oxidant effect. Conversely, Ce (2b′)_3_ at 3·10^−5^ M scavenges superoxide with CL-SI = 69 ± 3%.

Hypochlorous acid is part of the human body’s immune defense (Pattison and Davies, 2006), a product of the enzyme myeloperoxidase. It acts as a strong, oxidizing, non-specific bactericide, associated with multiple pathologies (Strzepa et al., 2017). The impact of 2b, La (2b′)_3_, and Ce (2b′)_3_ on LDCL in the presence of sodium hypochlorite is presented in Figure 9B. All three compounds behave as significant pro-oxidants. At 1·10^−4^ M, 2b has CL-SI = 140 ± 2% and at 3·10^−5^ M La (2b′)_3_ has CL-SI = 161 ± 2%. At the highest tested molarities, the effect of the La(III) complex (at 3·10^−5^ M) is higher than that of the ligand at three times the concentration (1·10^−4^ M). Considering that each complex bears three ligands, it seems that the La(III) ion potentiates the pro-oxidant behavior of 2b in this model system. Most striking are the results of the cerium complex. Pro-oxidant behavior is observed at molarities as low as 1·10^−7^ M (CL-SI = 115 ± 1%), increasing in a concentration-dependent manner to as high as CL-SI = 656 ± 21% at 3·10^−5^ M.

#### 3.5.6 Comparison between the activity of Ce (2b′)_3_ and Trolox

**FIGURE 10:**
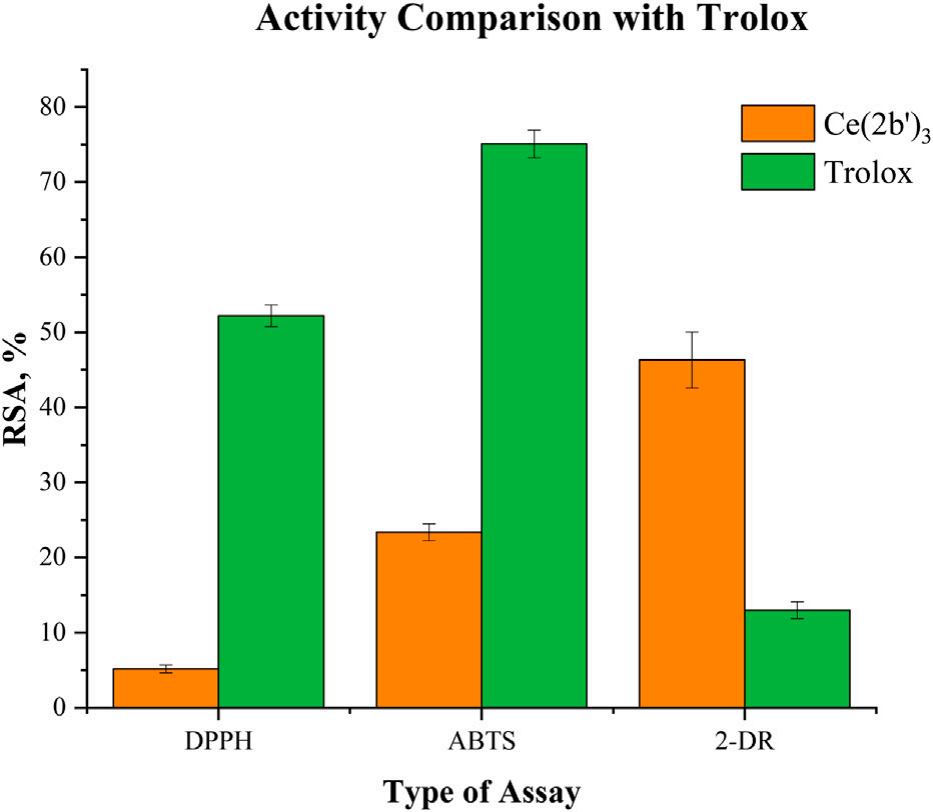
Comparison between Ce (2b′)_3_ and Trolox at 3·10^−5^ M in the DPPH, ABTS, and 2-deoxyribose (2-DR) model systems. Data = mean ± StDev, *p* < 0.05, N = 3. A higher result means higher scavenging activity.

A well-established positive control (Trolox) was tested at 3·10^−5^ M in order to compare its activity with the activity of the cerium complex at the same molarity. As Trolox does not dissolve well in bi-distilled water, PBS (pH = 7.45) was utilized to produce a clear 1·10^−3^ M stock solution. Since ferric ions form a precipitate in PBS, Trolox could not be tested in the Fenton reaction model system. Results obtained from the spectrophotometric assays are presented in Figure 10.

Compared to Trolox, the cerium complex manifests significantly lower DPPH scavenging activity (RSA = 52.2 ± 1.4% versus 5.2 ± 0.2%). A similar trend is observed in the ABTS assay: RSA = 75.1 ± 1.8% for Trolox and RSA = 23.4 ± 1.1% for Ce (2b′)_3_. Those results contrast with observations from the 2-deoxyribose degradation assay. OH^·^ scavenging activity of the complex in this model system is much higher than that of the positive control RSA = 46.3 ± 3.7% (complex) versus RSA = 13.0 ± 1.1% (Trolox).

At 3·10^−5^ M, the cerium complex performs as a moderate scavenger of superoxide in the KO_2_ model system (CL-SI = 69 ± 3%). At the same molarity, Trolox behaves as a much stronger scavenger (CL-SI = 25 ± 2%). When it comes to interaction with NaOCl, Ce (2b′)_3_ behaves as a very potent pro-oxidant CL-SI = 656 ± 21%, while Trolox almost totally extinguishes LDCL CL-SI = 1.5 ± 0.1%.

## 4 Summary and conclusion

A novel rare-earth metal ion complex with a biologically active ligand was synthesized, and its structure, electronic properties, and antioxidant activity were carefully studied by the experimental methods and quantum-mechanical calculations. The composition and structure of the tri-coordinated complex of Ce^3+^ and 2-(4-chlorophenyl)-5-(pyrrolidin-1-yl)-2*H*-1,2,3-triazole-4-carboxylate were determined using elemental analysis and spectral data. The structural features of Ce (2b′)_3_ and global chemical reactivity descriptors were revealed based on quantum chemical calculations using four DFT levels in comparison with three other models of cerium complexes.

The influence of different ligands on the charge and molecular properties of the Ce(III) complex was analyzed, and established and global reactivity descriptors were determined and demonstrated: 1) the better charge distribution on the cerium ion in larger systems; 2) the HOMO–LUMO gap value linearly decreases as system complexity increases with the exception of C complex that has a similar value to Ce (2b′)_3_. The low energy value indicates a large reactivity and low energy for excitation. 3) The analysis of the experimental and calculated IR and Raman spectra supported the proposed structure of the obtained Ce(III) complex. For the analysis of these spectra, detailed comparisons were carried out using mainly the M06-2X, CAM-B3LYP, and D3-B3LYP methods, obtaining the best results with the CAM-B3LYP method.

The complex Ce (2b′)_3_ scavenges hydroxyl radicals, generated by UV-induced water radiolysis to a greater extent than the positive control Trolox at 3·10^−5^ M. In this particular model system the type of coordination center, La(III) or Ce(III), has a very mild impact on activity. In line with previous observations on 2b and La (2b′)_3_, the ability of Ce (2b′)_3_ to participate in HAT with DPPH^·^ is very limited. These results, starkly contrasting with the results derived from the 2-deoxyribose degradation assay, suggest that the low DPPH-scavenging activity may be due to steric hindrance rather than the low hydrogen-donating capacity of the complex. Coordination with Ce(III) seems to improve the ligand’s ability to participate in SET, as observed in the ABTS^·+^ assay. Ce (2b′)_3_ seems to scavenge the stable radical much more actively compared to its La(III) counterpart (almost threefold higher RSA values). In this case, we can comfortably deduce that the electron-exchanging capacity is significantly impacted by the type of metal ion, which is coordinated with 2b. Ce (2b′)_3_ reaffirms previous observations that 2b and its lanthanide complexes tend to behave as pro-oxidants in the presence of the clinically significant Fenton reaction. A concentration-dependent increase in MTT-formazan formation was observed with Ce (2b′)_3_, which is similar to La (2b′)_3_. Ionic mimicry allows lanthanide ions to competitively replace iron from biomolecules (e.g., iron-dependent enzymes), impairing their functions and therefore, exhibiting chemotherapeutic effects. This would also cause an increase in “free” iron that can yield toxic OH^·^ via the Fenton reaction. On top of that, the abovementioned reaction seems to be enhanced in the presence of the ligand 2b both in free form and as a component of a complex. Unlike the pro-oxidant 2b and its La(III) complex, Ce (2b′)_3_ acts as an antioxidant in the presence of KO_2_. A very strong pro-oxidant effect of the Ce(III) complex is observed in the presence of sodium hypochlorite, an RS that serves as a component of the body’s immune defense against xenobiotics. The present research introduces to the reader a second biologically active lanthanide complex with 2b. Both La(III) and Ce(III) complexes manifest interesting biological behavior, serving as antioxidants in some model systems and as antioxidants in others. Additionally, the type of the lanthanide coordination center (in this case Ce^3+^ instead of La^3+^) was associated with a change in biological behavior, which is observed prominently in the ABTS, KO_2_, and NaOCl assays. The most logical next steps were left for further research, which involve investigations on other lanthanide complexes of 2b, selecting those with most prominent activities and consequently testing them against bacterial/cancer cell lines. The results presented herein could lay a solid foundation for the synthesis of additional, novel lanthanide–triazole carboxylate complexes with promising biological activity. For this task, the relationships established could facilitate the selection of new ligands with improved properties in this design of new complexes.

## Data Availability

The original contributions presented in the study are included in the article/Supplementary Material; further inquiries can be directed to the corresponding authors.
