# Supplementary material for: Molecular properties of a triazole–Ce(III) complex with antioxidant activity: structure, spectroscopy, and relationships with related derivatives. Influence of the ligands in the complex
Source: Front Chem. 2024 Nov 6;12:1450106. doi: 10.3389/fchem.2024.1450106 (PMC11576285; doi:10.3389/fchem.2024.1450106)
Supplement: Supplementary file 1 [file DataSheet1.PDF]

Frontiers in Chemistry

SUPPLEMENTARY MATERIAL

Molecular Properties of a Triazole-Ce(III) Complex with Antioxidant Activity: Structure, Spectroscopy and Relationships with related derivatives. Influence of the ligands in the complex

M. Alcolea Palafox[1*], Nataliya P. Belskaya[2], Lozan Todorov [3*], Nadya Hristiva-Avakumova[4] and Irena P. Kostova [3]

[1] *Departamento de Química Física, Facultad de Ciencias Químicas, Universidad Complutense, Madrid-28040, Spain* (alcolea@ucm.es)

[2] *Department of Technology for Organic Synthesis, Ural Federal University, 19 Mira Str., Yekaterinburg 620012, Russia;* (n.p.belskaya@urfu.ru)

[3] *Department of Chemistry, Faculty of Pharmacy, Medical University – Sofia, 2 Dunav Str., 1000 Sofia, Bulgaria*

[4] *Department of Medical Physics and Biophysics, Faculty of Medicine, Medical University of Sofia, 2 Zdrave Str., 1431 Sofia, Bulgaria*

* Correspondence: L.T.: alcolea@ucm.es; ltodorov@pharmfac.mu-sofia.bg

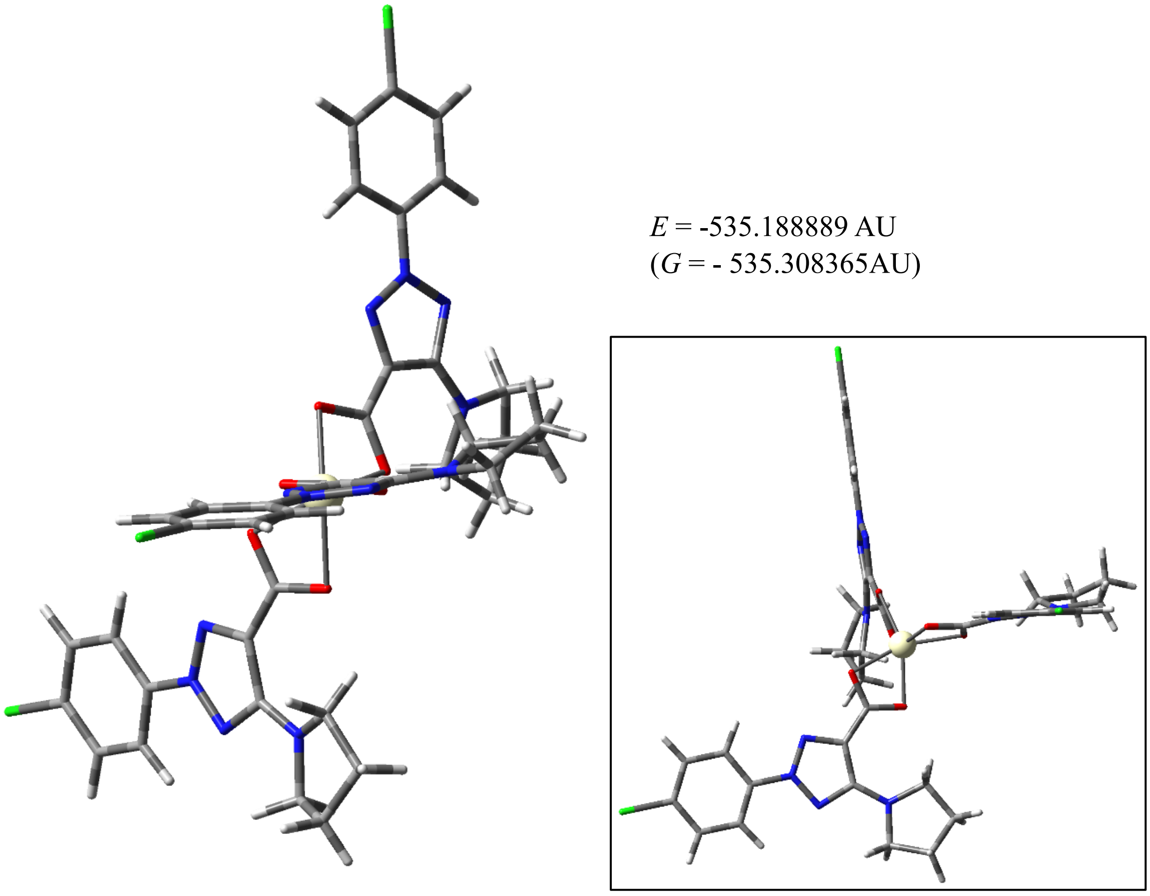

Fig. S1. Plot of the optimized Ce(**2b′**)$_3$ structure with **2b′**: sodium 2-(4-chlorophenyl)-5-(pyrrolidin-1-yl)-2H-1,2,3-triazole-4-carboxylate at the B3LYP/Cep-4g level.

D3-B3LYP/Cep-4g

E = -535.396203 AU   (G = -535.516241 AU)

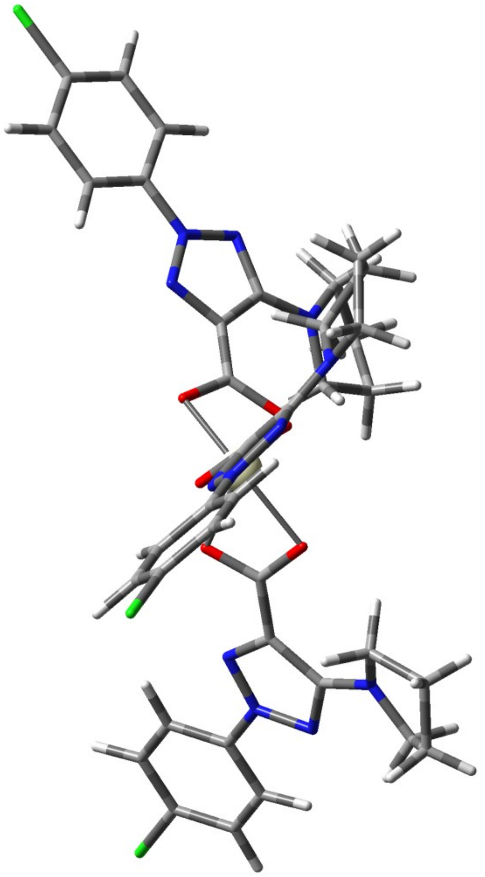

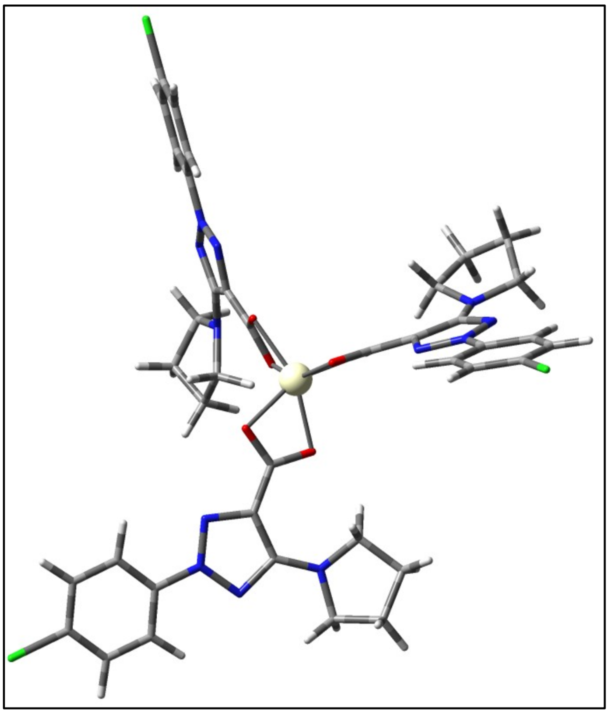

Fig. S2. Plot of the optimized Ce(**2b′**)$_3$ structure with **2b′**: sodium 2-(4-chlorophenyl)-5-(pyrrolidin-1-yl)-2*H*-1,2,3-triazole-4-carboxylate at the D3-B3LYP/Cep-4g level.

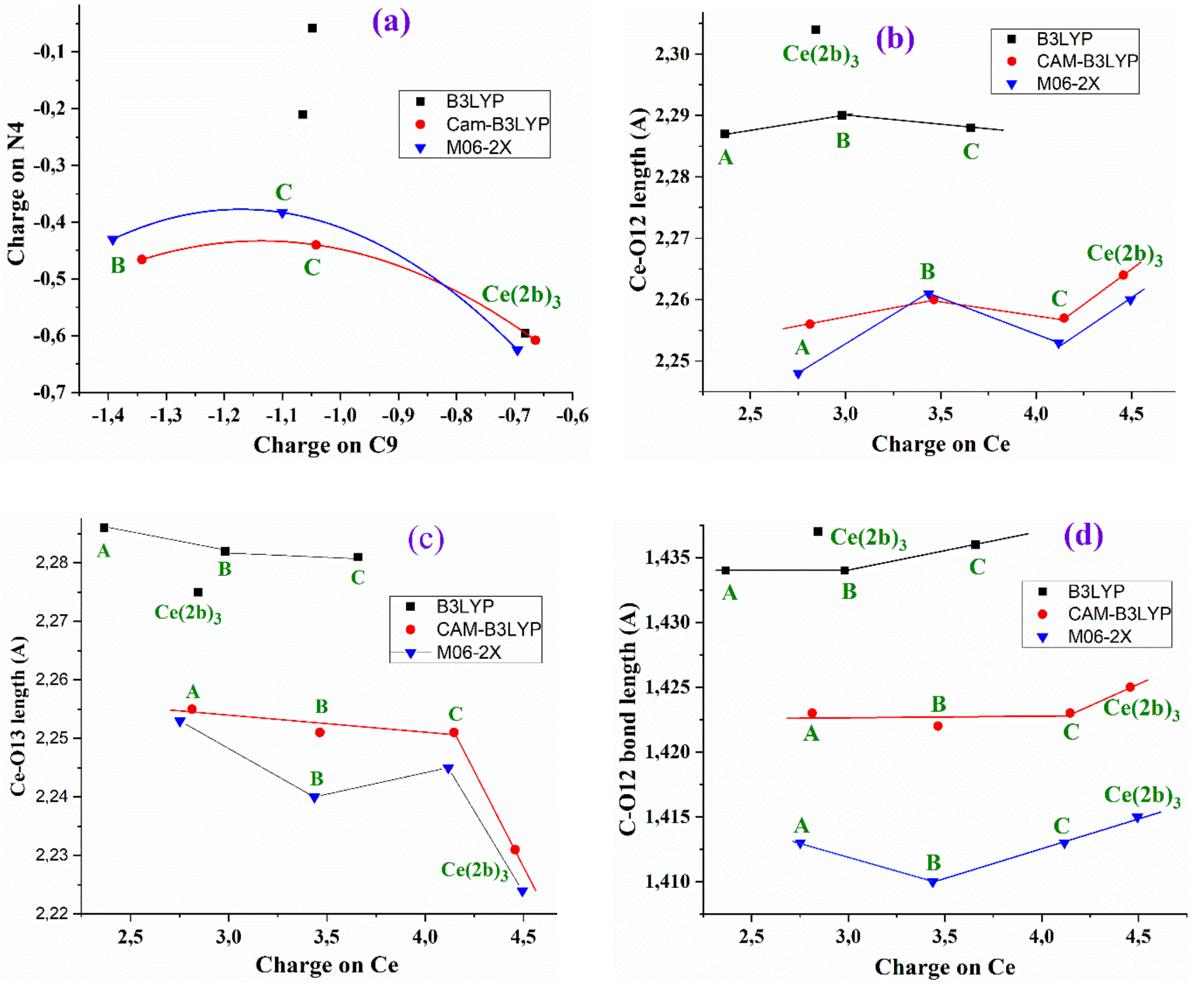

Fig. S3. Relationships with the B3LYP, CAM-B3LYP and M06-2X methods between the positive calculated atomic APT charge on the cerium atom in the A-, B-, C- and Ce(2b′)$_3$ complexes

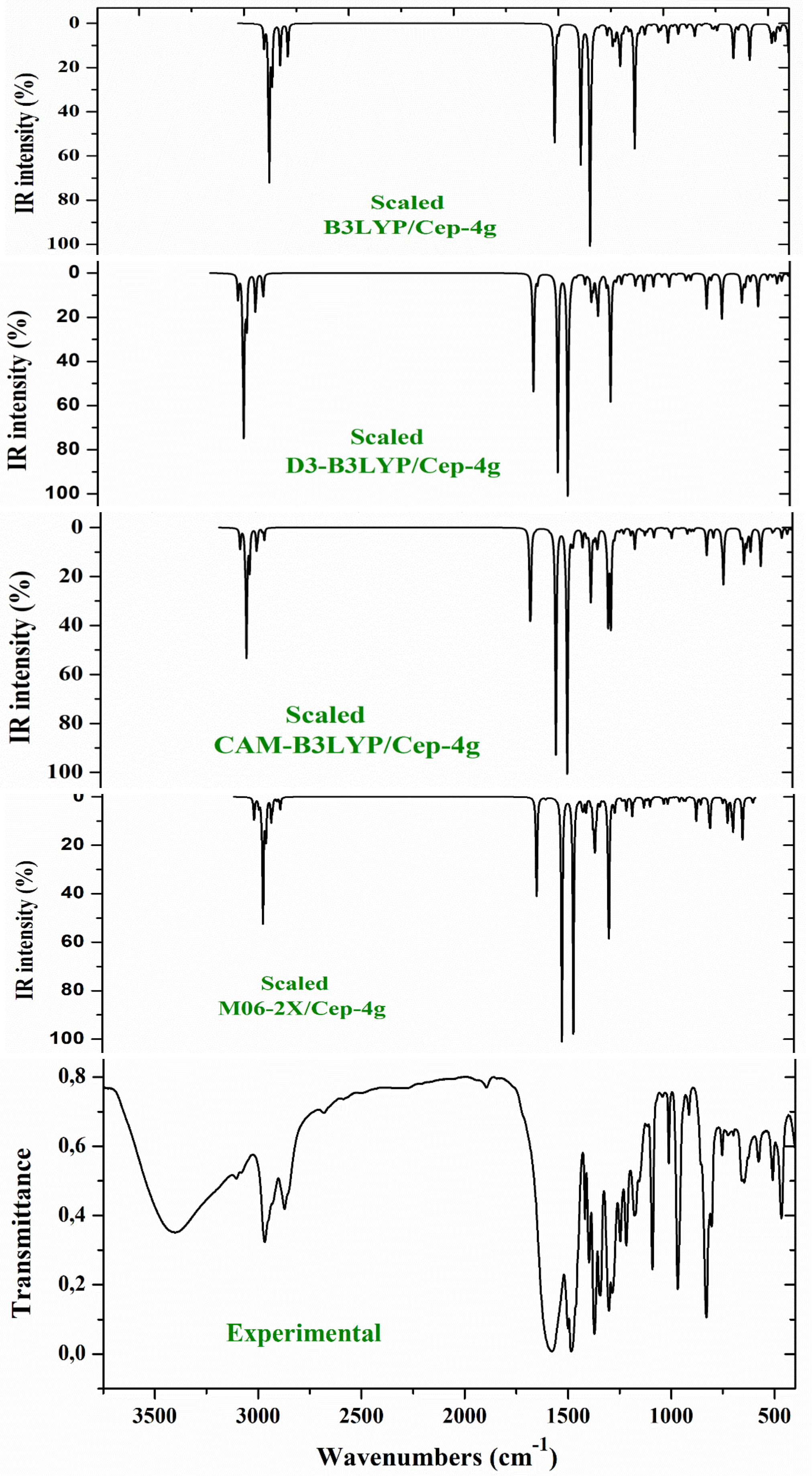

Fig. S4. Comparison of the theoretical scaled IR spectra with the experimental ones in the 3750-400 cm^{-1} range.

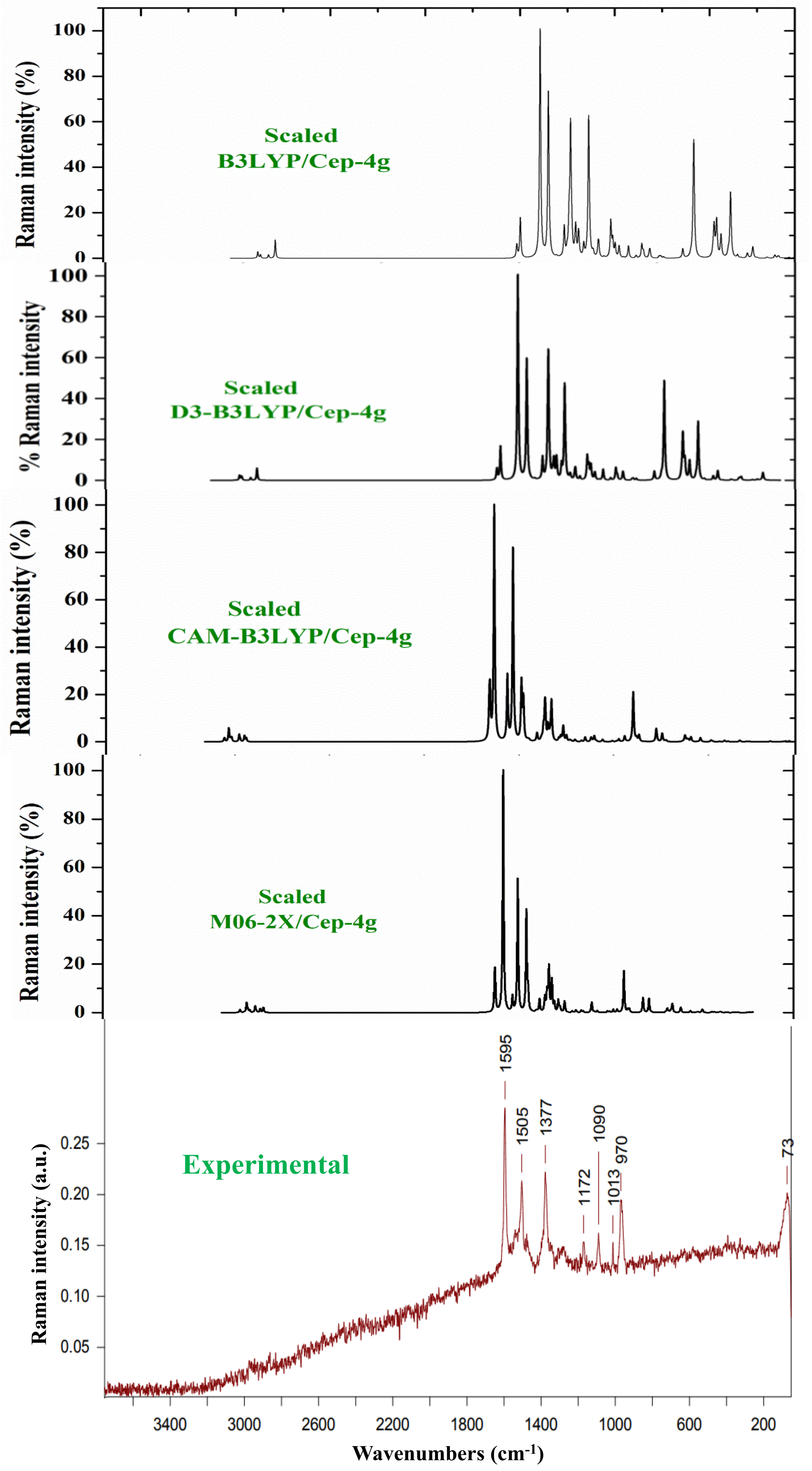

Fig. S5. Comparison of the theoretical scaled Raman spectra with the experimental ones in the 3750-0 cm^{-1} range.

Table S1. Calculated, scaled and experimental wavenumbers (ν, cm^{-1}) in the Ce(2b′)$_3$ complex with CAM-B3LYP method. Relative infrared intensity (A) in %, relative Raman intensity (S) in %, and Raman depolarization ratios for plane (DP) and unpolarized incident light (DU). For each vibration of the tetramer, the wavenumber with the highest IR intensity is indicated in bold type and that with the highest Raman intensity is indicated in italic type. The relative IR and Raman intensities were shown only for these wavenumbers. DP and DU values were from most intense Raman line. The number of the ring mode corresponds to Wilson's notation (Varsányi, 1974).

Calculated by **CAM-B3LYP**					scaled		Experimental		
ν	A	S	DP	DU	LSE	PSE	IR	Raman	Characterization
	--	--					3400.8 br, s		ν(O-H) H_2O bonded
3364, 3364, 3364	8	2	0.73	0.84	3093	3074			ν_{as}(C-H) in $C16H_2$, $C17H_2$ (100)
3361, 3360, 3360	3	2	0.75	0.86	3089	3071			ν_{as}(C-H) in $C16H_2$, $C17H_2$ (100)
3333, 3333, 3333	3	1	0.20	0.33	3066	3050			ν_{as}(C-H) in $C18H_2$, pyrrolidine (100)
3332, 3331, 3331	4	11	0.00	0.01	3064	3049			2, ν(C3-H) in aryl (100)
3324, 3323, 3323	47	1	0.70	0.82	3057	3043	2968.1 s		20b, ν(C5-H) in aryl (100)
3317, 3317, 3317	7	1	0.75	0.86	3052	3038			ν_{as}(C-H) in $C15H_2$ in pyrrolidine (100)
3312, 3312, 3312	4	3	0.64	0.78	3048	3034			7b, ν(C6-H) in aryl (100)
3303, 3303, 3303	13	0	0.75	0.86	3040	3028			20a, ν(C2-H) in aryl (100)
3260, 3260, 3260	8	10	0.01	0.02	3003	2995			ν_s(C-H) in $C16H_2$, $C17H_2$ (100)
3253, 3253, 3253	4	1	0.75	0.86	2997	2989			ν_s(C-H) in $C16H_2$, $C17H_2$ in pyrrolidine (100)
3224, 3224, 3224	1	7	0.03	0.06	2971	2967			ν_s(C-H) in $C18H_2$ in pyrrolidine (100)
3211, 3211, 3211	5	3	0.01	0.02	2960	2957	2872.1 m		ν_s(C-H) in $C15H_2$ in pyrrolidine (100)
	--	--	-	-			1894.9 vw		Combination band
1664, 1660, 1660	56	35	0.09	0.16	1623	1671	1577.2 br, vs		$\nu(C_8-N_{14})$ (45) + ν_s(CC) (34)
1644, 1644, 1644	0	0	0.22	0.36	1605	1653			8b, ν(C=C) in aryl (89)
1635, 1635, 1635	0	92	0.18	0.31	1598	1645		1595.0 vs	8a, ν(C=C) in aryl (82)
1558, **1525**, *1525*	97	100	0.75	0.86	1503	1546	1500.1 s	1504.5 s	ν_{as}(COO)+$\nu(C_9-C_{11})$+$\nu(C_4N)$+19a,ν(CC,CH)
1475, **1464**, 1464	100	60	0.04	0.08	1450	1491	1484.4 vs		19a,ν(CC,CH)+$\nu(C_9-C_{11})$+ν(triazol)
1461, 1461, 1461	6	1	0.74	0.85	1447	1488			δ(C-H) in-phase in pyrrolidine (92)
1440, 1440, 1440	4	0	0.75	0.86	1429	1469			δ(CH) in pyrrolidine (95)
1434, 1433, 1433	5	0	0.75	0.86	1423	1463			δ_s(C-H) out-of-phase in pyrrolidine (88)
1429, 1429, 1429	1	0	0.23	0.37	1420	1459			δ_s(C-H) out-of-phase in pyrrolidine (85)
1385, 1383, 1383	7	7	0.04	0.07	1380	1417	1418.0 w		ν_{as}(NNN, CN) +19a,ν(CC)(35)+ν_s(COO)
1362, 1362, 1362	2	1	0.75	0.86	1362	1398	1398.3 m		19b, ν(CC)(85)
-	--	--					-	-	-
1357, 1355, 1355	1	1	0.50	0.67	1356	1391			δ_s(C-H) pyrrolidine +19a,ν(CC)+ν(triazol)
1347, **1339**, 1339	26	12	0.10	0.18	1342	1377	1372.2 vs	1376.8 vs	ν_s(COO)+ν(triazol)+3,δ(CH)+δ(pyrrolidine)
1337, 1336, 1336	7	16	0.05	0.09	1339	1374			ν_s(COO) (49) +δ(pyrrolidine)+ ν(triazol)
1321, 1321, 1321	2	6	0.03	0.07	1326	1360			δ(C-H) in pyrrolidine +3, δ(C-H) +ν(COO)
1313, 1309, **1309**	2	3	0.14	0.25	1316	1349			δ(C-H) in pyrrolidine +ν_{as}(COO)
1306, 1305, 1305	6	1	0.75	0.86	1312	1345	1343.1 s		δ_s(C-H) in pyrrolidine +ν_{as}(COO)
1303, 1301, *1301*	3	19	0.75	0.86	1309	1342			ν(CO12)+3, δ(C-H) aryl+δ(triazol)+δ(pirrol)
1259, **1248**, 1248	40	3	0.35	0.52	1263	1293	1301.8 vs		ν(triazol) + ν_{as}(CO13) + Γ(pyrrolidine)
1238, *1234*, **1233**	41	7	0.75	0.86	1250	1279	1285.1 s		ν_{as}(CO13) + ν(C-N) triazol +Γ(pyrrolidine)
1216, 1215, 1215	3	3	0.75	0.86	1234	1262			ν(CO12) + ν(C-N) triazol +δ(C-H) in aryl
1197, 1197, 1197	0	1	0.75	0.86	1219	1246			δ(C-H) in pyrrolidine
1189, 1188, 1188	1	0	0.75	0.86	1211	1237	1246.8 m		δ_s(C-H) in pyrrolidine
1168, 1168, 1168	2	1	0.75	0.86	1194	1219			3, δ(C-H) in aryl
	--	--							
1163, 1163, 1163	1	0	0.75	0.86	1190	1214	1218.1 m		γ_{as}(CH) in pyrrolidine
1131, 1130, 1130	3	1	0.75	0.86	1161	1183	1178.3 m		γ_{as}(CH) in pyrrolidine + ν_s(NNN)
1112, 1109, ***1109***	9	2	0.75	0.86	1143	1164		1171.5 w	ν_s(COO) + ν(NCCN) + γ_s(CC,CH)
1075, 1075, 1075	1	2	0.02	0.04	1113	1132	1150 sh		9a, δ(C-H) in aryl
1068, 1067, 1067	1	0	0.01	0.02	1107	1124			γ(C-H) in pyrrolidine (73)
1057, 1057, 1057	3	3	0.02	0.03	1098	1115			1, δ(CCC) in aryl
1046, 1045, 1045	1	0	0.75	0.85	1088	1104			δ(C-C) in pyrrolidine
1016, 1015, 1015	1	0	0.75	0.86	1062	1075			14, ν(CC) in aryl
1011, 1010, 1010	4	1	0.75	0.86	1057	1071	1091.2 vs	1089.9 m	ν(triazol)+ν(C-CL)+ν_s(COO)+18a,δ(CC,CH)
956, 956, 956	0	0	0.73	0.84	1011	1020	1011.9 m	1012.8 w	γ_{as}(CC,CH) in pyrrolidine
932, 931, 931	1	0	0.09	0.16	989	996	969.1 vs		ν_{as}(triazol) + ν_s(COO) + γ(CC,CH)
920, 920, 919	4	1	0.75	0.86	979	986			18a, δ(C-H) +ν(NN,CN) +γ(pyrrolidine)
887, 887, 887	0	4	0.02	0.04	951	954			γ(CC) pyrrolidine +ν_{as}(NNN)
878, 878, 878	0	0	0.66	0.80	943	946			γ(CC) pyrrolidine

852, 852, 852	0	0	0.20	0.34	921	921			17a, γ(C-H)
842, 842, 842	0	0	0.75	0.86	912	911			5, γ(C-H) in aryl
840, 839, 839	4	22	0.04	0.08	909	908	914.3 w	970.2 s	ν_{as}(NNN) + 12, δ(CCC) in aryl
821, 821, 821	1	2	0.10	0.18	894	891			γ_{as}(C-C) in pyrrolidine
808, 808, 808	1	3	0.08	0.14	883	879			γ_{as}(C-H) in pyrrolidine + ν_{as}(NNN) (12)
771, 771, 771	0	0	0.63	0.78	851	843			10a, γ(C-H) in aryl
742, 742, 742	9	0	0.75	0.86	826	815	857 w-m		17b, γ(C-H) in aryl
713, **709**, 709	4	11	0.07	0.13	797	783	829.9 vs		γ_s(COO) + ν(triazol) + γ(CC) pyrrolidine
680, **667**, 667	4	7	0.06	0.12	761	743	804.8 m		δ_{as}(COO) + 6a, δ(CCC) +δ_{as}(NNN)
665, 659, ***658***	24	1	0.75	0.86	753	734	754.1 w		δ_s(COO) + δ(triazol) +γ(CC) pyrrolidine
572, **572**, *568*	3	0	0.04	0.07	679	650	699.2 vw		γ_s(COO) + γ(C9-C11)
567, ***557***, 557	12	2	0.75	0.86	666	636	654 m		γ(triazol) + δ_{as}(COO) + 4,γ(CCC)
555, 554, 554	4	1	0.75	0.86	663	633	647.1 m		Γ(triazol) + δ_{as}(COO)+ 4,γCCC)
546, 544, ***544***	6	1	0.75	0.86	654	623			6b, δ(CCC) +δ(COO) + γ(triazol)
540, 539, 539	1	0	0.38	0.55	650	618			γ_{as}(C-H) in pyrrolidine + 6b, δ(CCC) in aryl
537, 537, 537	1	0	0.74	0.85	648	616			γ_{as}(C-H) in pyrrolidine + δ(triazol)
526, 524, ***524***	10	2	0.75	0.86	637	603	576.8 w		γ(triazol) + γ(COO) + 6b, δ(CCC) in aryl
493, 493, 493	0	0	0.75	0.86	610	573			γ(triazol) +16b, γ(CCC) in aryl
474, 474, 473	16	2	0.05	0.10	594	554	509.0 m		δ_{as}(COO) + δ(triazol) + δ(CC) in aryl
418, 416, ***416***	2	1	0.75	0.86	544	497			δ_{as}(COO) +δ(triazol) + δ(CC) in aryl
408, 408, 408	1	0	0.75	0.86	537	489			16b, γ(CCC) + γ(triazol) + δ(COO)
371, ***371***, 369	4	0	0.75	0.85	505	452	466.9 m		ν(aryl,C-CL) + δ(COO) + δ(triazol)
344, 344, 343	3	1	0.75	0.86	481	426			δ(COO) + δ(triazol) + ν(aryl,C-CL)
335, 335, 335	0	0	0.75	0.85	474	417			16a, δ(CCC)
								73.1 br m	

Table S2. Vibrational assignment with the M06-2X method.

Calculated by **M06-2X**			scaled		Experimental		Characterization
ν	A	S	LSE	PSE	IR	Raman	
					3400.8 br, s		ν(O-H) H_2O bonded
3380, 3379, 3379	6	1	3095	3076			ν_{as}(C-H) in C16H$_2$, C17H$_2$ (100)
3376, 3376, 3376	2	1	3093	3074			ν_{as}(C-H) in C16H$_2$, C17H$_2$ (100)
3351, 3350, 3350	3	0	3070	3054			ν_{as}(C-H) in C18H$_2$ pyrrolidine
3334, 3334, 3333	3	7	3057	3042			2, ν(C_3-H) in aryl (100)
3327, 3326, 3326	5	1	3050	3036			ν_{as}(C-H) in C15H$_2$ pyrrolidine (100)
3326, 3326, 3325	37	1	3050	3036	2968.1 s		7b, ν(C_5-H) in aryl (100)
3314, 3314, 3313	3	1	3039	3027			20b, ν(C_6-H) in aryl (100)
3308, 3308, 3307	15	0	3034	3022			20a, ν(C_2-H) in aryl (100)
3277, 3277, 3276	8	6	3007	2999			ν_s(C-H) in C17H$_2$, C16H$_2$ pyrrolidine (100)
3269,3269, 3268	3	1	3001	2993			ν_s(C-H) in C17H$_2$, C18H$_2$ pyrrolidine (100)
3244, 3244, 3243	1	2	2979	2974			ν_s(C-H) in C18H$_2$ pyrrolidine (100)
3223, 3223, 3223	6	5	2961	2957	2872.1 m		ν_s(C-H) in C15H$_2$ pyrrolidine (100)
-	--	--	--	--	1894.9 vw		Combination band
1702, **1700**, 1698	46	20	1649	1699	1577.2 br, vs		ν(C_8-N_{14}) + ν_s(NNN-NC) in triazol
1652, 1651, 1651	0	1	1607	1655			8b, ν(C=C) in aryl (93)
1646, 1646, 1645	1	100	1603	1651		1595.0 vs	8a, ν(C=C) in aryl (93)
1584, ***1550***, 1549	100	73	1520	1565	1500.1 s	1504.5 s	ν_{as}(COO) + ν(C_9-C_{11}) + ν_s(CCN) triazol + ν(C_4-N_4)
1493, 1482, **1481**	94	98	1461	1503	1484.4 vs		19a, ν(CC,CH) + ν(C_4-N_4) + ν(C_9-C_{11})
1452, 1452, 1452	1	0	1436	1477			δ(C-H) in pyrrolidine
1432, 1431, 1431	2	1	1418	1458			δ(C-H) in pyrrolidine
1426, 1426, 1425	3	0	1413	1453			δ_s(C-H) in pyrrolidine
1421, 1421, 1420	3	1	1409	1448			δ_s(C-H) in pyrrolidine (85)
1407, **1405**, 1405	5	10	1395	1434	1418.0 w		ν_{as}(NNN) in triazol + 19a,ν(CC) in aryl
1378, 1378, 1378	2	0	1372	1409	1398.3 m		19b, ν(CC,CH) in aryl
1371, 1365, **1365**	9	13	1361	1397	-	-	δ_s(C-H) in pyrrolidine + δ(C_{14}-N_{14})
1358, 1357, **1352**	28	9	1349	1386	1372.2 vs	1376.8 vs	ν_s(COO) + ν_s(C_9-N_{10}) triazol + 19b, ν(CC,CH) in aryl
1345, 1344, 1344	8	19	1343	1378			ν_{as}(NNN) + ν_{as}(COO) + 19a,ν(CC) + δ(CC) pyrrolidine
1330, **1325**, *1325*	2	14	1326	1361			ν_{as}(COO) + ν_{as}(triazol) + ν_{as}(CNC) pyrrol + 19a,ν(CC)
1323, 1323, 1322	1	1	1324	1359			δ_s(C-H) in pyrrolidine + ν_s(NNN)+ν_{as}(COO)+19a,ν(CC)
-	--	--	--	--	-	-	-
1309, 1308, 1308	0	4	1312	1345	1343.1 s		δ_s(C-H) in pyrrolidine + ν_s(NNN)
1307, 1307, 1306	0	1	1311	1344			δ_s(C-H) in pyrrolidine
1283, 1274, **1269**	81	11	1278	1309	1301.8 vs		ν(triazol) (45) + ν(CO_{13})(40) + δ_s(CC,CH) pyrrolidine (12)
1242, 1242, 1241	1	4	1255	1285	1285.1 s		ν_{as}(CO_{12}) + ν_s(C_9NN) triazol + δ_s(CC,CH) in pyrrolidine

1236, 1235, 1235	5	0	1249	1278			Γ(C-H) in pyrrolidine + ν_s(C_9NN) triazol
1193, 1192, 1192	1	1	1212	1238	1246.8 m		δ_s(C-H) in pyrrolidine
1184, 1184, 1183	1	0	1205	1231			γ_{as}(C-H) in pyrrolidine
1168, **1166**, 1166	5	1	1189	1214	1218.1 m		γ_{as}(C-H) in pyrrolidine + 3, δ(CH) + ν_s(NNN)
1162, 1162, 1161	0	0	1186	1211			3, δ(CH) + ν_{as}(NNN) + γ_{as}(C-H) in pyrrolidine
1134, 1132, ***1131***	8	1	1159	1182			ν_s(COO) + ν(triazol) + δ_{as}(C-H) in pyrrolidine
-	-	-	-	-	-	-	-
1119, 1119, 1118	1	1	1149	1170	1178.3 m	1171.5 w	γ_{as}(C-H) in pyrrolidine + ν_s(triazol)
1073, 1073, 1073	0	0	1109	1128	1150 sh		9a, δ(C-H) in aryl
1066, 1065, 1065	0	1	1102	1120			δ_{as}(C-H, CC) in pyrrolidine + 9a, δ(C-H) in aryl
1063, 1062, 1062	4	4	1100	1117			18a, δ(C-H) in aryl
1046, 1045, 1045	1	1	1085	1101			δ_{as}(C-H, CC) in pyrrolidine
1027, 1025, ***1025***	4	1	1068	1083	1091.2 vs	1089.9 m	ν_s(NNN) + ν(C-CL) +18a, δ(C-H) in aryl
1013, 1013, 1012	0	0	1057	1071			18b, δ(C-H) in aryl
959, 958, 958	0	1	1010	1019	1011.9 m	1012.8 w	γ_{as}(CC, CH) in pyrrolidine
945, 944, 944	3	1	998	1006			ν_{as}(NC_9-C_8) + δ_{as}(CC, CH) in pyrrolidine
921, 920, 920	3	1	977	983	969.1 vs		ν_{as}(NNN) + 18a, δ(C-H) in aryl
896, 896, 896	0	1	957	961			γ_s(C-H) in pyrrolidine
881, 881, 880	0	0	944	946			γ_{as}(C-H) in pyrrolidine
866, 866, 866	0	0	931	932			17a, γ(C-H) in aryl
854, 854, 853	0	0	921	921			5, γ(C-H) in aryl
851, 851, 851	2	19	918	918		970.2 s	ν_{as}(NNN) (36) + 12, δ(CCC) in aryl
826, 825, 825	1	1	896	893	914.3 w		γ_{as}(CC, CH) pyrrolidine
815, 815, 814	1	1	887	883			γ_{as}(CC, CH) pyrrolidine
775, 775, 774	0	0	853	845			10a, γ(C-H) in aryl
							-
751, 750, 750	7	0	831	821	857 w-m		17b, γ (C-H) in aryl
725, 724, **723**	3	10	808	795	829.9 vs		γ_s(COO) + γ(CC, CH) pyrrolidine + δ(triazol)
686, **674**, 674	7	11	766	748	804.8 m		δ_{as}(COO) + 6a, δ(CC) in aryl
671, 668, **668**	10	0	760	742	754.1 w		δ_s(COO) + δ_{as}(CC) pyrrolidine + 6a, δ(CC) in aryl
**594**, *593*, 589	2	0	697	670	699.2 vw		γ(COO) + γ(C_9-C_{11})
575, 573, 573	0	1	680	652	654 m		γ(triazol) + δ_{as}(COO) + 8b, δ(CCC) in aryl
572, 565, ***564***	11	2	671	641	647.1 m		Γ(triazol) + δ_{as}(COO)
555, 554, 553	0	0	663	632			γ(triazol) + γ_{as}(COO)
544, ***541***, 540	5	1	651	619			γ(triazol) + γ_{as}(COO) + 6b, δ(CCC) in aryl
538, 537, 537	1	0	648	615			γ_{as}(C-H) in pyrrolidine
535, 532, ***531***	14	4	642	609	576.8 w		6a, δ(CCC) + γ(triazol) + δ(COO)
524, 523, 523	0	0	635	601			γ_s(NNN) +18b, γ(CCC) in aryl
478, 476, **475**	16	2	594	554	509.0 m		δ_{as}(COO) + δ(triazol) + δ(CC) in aryl
**419**, *418*, 417	1	0	546	499			16b, γ(CCC) in aryl
416, ***413***, 412	3	1	541	493			γ(CC) in aryl + γ(triazol) + δ_{as}(COO)
372, 370, ***363***	4	1	498	443	466.9 m		ν(aryl, C-CL) + δ_{as}(COO) + δ(triazol)
						73.1 br m	
